# The MocR/GabR Ectoine and Hydroxyectoine Catabolism Regulator EnuR: Inducer and DNA Binding

**DOI:** 10.3389/fmicb.2021.764731

**Published:** 2021-12-24

**Authors:** Lucas Hermann, Felix Dempwolff, Wieland Steinchen, Sven-Andreas Freibert, Sander H. J. Smits, Andreas Seubert, Erhard Bremer

**Affiliations:** ^1^Faculty of Biology, Philipps-University Marburg, Marburg, Germany; ^2^Department of Biochemistry and Synthetic Metabolism, Max-Planck-Institute for Terrestrial Microbiology, Marburg, Germany; ^3^SYNMIKRO Research Center, Philipps-University Marburg, Marburg, Germany; ^4^Department of Medicine, Institute for Cytobiology and Cytopathology, and SYNMIKRO Research Center, Philipps-University Marburg, Marburg, Germany; ^5^Institute of Biochemistry, Heinrich-Heine-University, Düsseldorf, Germany; ^6^Center for Structural Studies (CSS), Faculty of Biochemistry, Heinrich-Heine-University, Düsseldorf, Germany; ^7^Faculty of Chemistry, Philipps-University Marburg, Marburg, Germany

**Keywords:** ectoine, hydroxyectoine, GntR transcription factor, repressor, inducer, PLP

## Abstract

The compatible solutes ectoine and 5-hydroxyectoine are widely synthesized by bacteria as osmostress protectants. These nitrogen-rich tetrahydropyrimidines can also be exploited as nutrients by microorganisms. Many ectoine/5-hydroxyectoine catabolic gene clusters are associated with a regulatory gene (*enuR*: ectoine nutrient utilization regulator) encoding a repressor protein belonging to the MocR/GabR sub-family of GntR-type transcription factors. Focusing on EnuR from the marine bacterium *Ruegeria pomeroyi*, we show that the dimerization of EnuR is mediated by its aminotransferase domain. This domain can fold independently from its amino-terminal DNA reading head and can incorporate pyridoxal-5′-phosphate (PLP) as cofactor. The covalent attachment of PLP to residue Lys302 of EnuR was proven by mass-spectrometry. PLP interacts with system-specific, ectoine and 5-hydroxyectoine-derived inducers: alpha-acetyldiaminobutyric acid (alpha-ADABA), and hydroxy-alpha-acetyldiaminobutyric acid (hydroxy-alpha-ADABA), respectively. These inducers are generated in cells actively growing with ectoines as sole carbon and nitrogen sources, by the EutD hydrolase and targeted metabolic analysis allowed their detection. EnuR binds these effector molecules with affinities in the low micro-molar range. Studies addressing the evolutionary conservation of EnuR, modelling of the EnuR structure, and docking experiments with the inducers provide an initial view into the cofactor and effector binding cavity. In this cavity, the two high-affinity inducers for EnuR, alpha-ADABA and hydroxy-alpha-ADABA, are positioned such that their respective primary nitrogen group can chemically interact with PLP. Purified EnuR bound with micro-molar affinity to a 48 base pair DNA fragment containing the sigma-70 type substrate-inducible promoter for the ectoine/5-hydroxyectoine importer and catabolic gene cluster. Consistent with the function of EnuR as a repressor, the core elements of the promoter overlap with two predicted EnuR operators. Our data lend themselves to a straightforward regulatory model for the initial encounter of EnuR-possessing ectoine/5-hydroxyectoine consumers with environmental ectoines and for the situation when the external supply of these compounds has been exhausted by catabolism.

## Introduction

One cornerstone of the evolutionary success of microorganisms is their enormous metabolic potential, a trait which allows them to take advantage of a wide spectrum of nutrients present in their varied ecological niches. To preserve precious energetic and biosynthetic resources, microorganisms exert a tight control over the expression of genes encoding nutrient uptake and utilization systems. In this process, activator or repressor proteins affecting transcription play a key role (Bervoets and Charlier, 2019). One of these are GntR-type transcription factors (Rigali et al., 2002; Jain, 2015; Vigouroux et al., 2021).

GntR family proteins possess a common domain-based architecture with an N-terminal DNA-reading head that typically contains a winged helix-turn-helix operator binding motif and a C-terminal oligomerization and effector-binding domain. Depending on the type and fold of the C-terminal domain, GntR-type transcription factors can be divided into several sub-families (Rigali et al., 2002; Jain, 2015); one of them is formed by MocR/GabR-type proteins (Rossbach et al., 1994; Bramucci et al., 2011; Suvorova and Rodionov, 2016; Tramonti et al., 2018; Pascarella, 2019). The genetically, biochemically, and structurally best characterized member of this sub-family is the GabR protein from *Bacillus subtilis*, a regulatory protein involved in the utilization of γ-amino-butyric acid (GABA) as a nitrogen source (Belitsky and Sonenshein, 2002; Belitsky, 2004; Edayathumangalam et al., 2013; Wu et al., 2017; Nardella et al., 2020).

The C-terminal effector-binding and oligomerization domains of MocR/GabR-type proteins resemble in their fold that of aminotransferases of type I, enzymes that depend on the cofactor pyridoxal-5′-phosphate (PLP, vitamin B6) for their activity (Percudani and Peracchi, 2003; Belitsky, 2004; Suvorova and Rodionov, 2016; Tramonti et al., 2018; Richts et al., 2019). However, the aminotransferase domain (ATD) of these regulatory proteins does not possess enzymatic activity; instead it is used as a sensory domain to affect DNA-binding in response to environmental or cellular cues (Percudani and Peracchi, 2003; Okuda et al., 2015b; Wu et al., 2017; Tramonti et al., 2018).

In some MocR/GabR regulators, in particular those that are involved in the synthesis of vitamin B6, a PLP molecule serves as the sole effector molecule (Belitsky, 2014; Richts et al., 2019). In other MocR/GabR-type regulators, the covalently bound PLP interacts chemically with system-specific low molecular mass inducer molecules (Edayathumangalam et al., 2013; Okuda et al., 2015b; Tramonti et al., 2018). Stemming from this interaction, internal and external aldimines are formed, thereby triggering a conformational change that affects the DNA-binding properties of the transcription factor (Edayathumangalam et al., 2013; Okuda et al., 2015b; Wu et al., 2017; Tramonti et al., 2018; Frezzini et al., 2020). The term internal aldimine refers to a PLP molecule covalently bound via a Schiff-base to the side chain of a lysine residue. This aldimine bond is hydrolyzed upon the chemical interaction of a low molecular mass inducer with the bound PLP molecule, a reaction that leads to the formation of a PLP: inducer adduct, the external aldimine (Tramonti et al., 2018).

**FIGURE 1:**
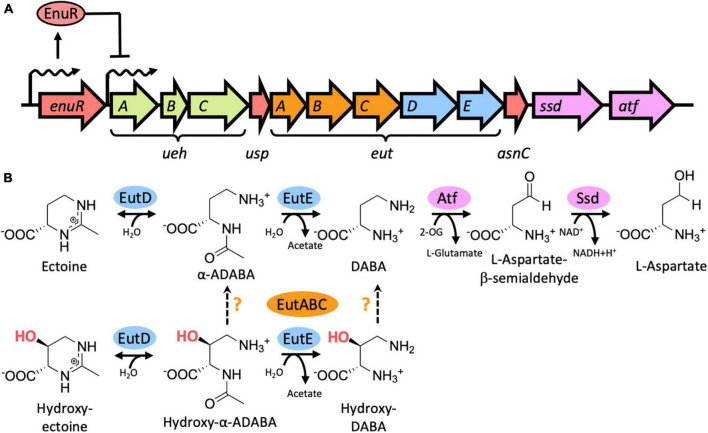
Genetic organization of the ectoine/5-hydroxyectoine importer and catabolic gene cluster from *Ruegeria pomeroyi* DSS-3, EutD/EutE-mediated catabolism of ectoine and 5-hydroxyectoine, and transcriptional induction of the ectoine/5-hydroxyectoine importer and catabolic genes. **(A)** Schematic overview of the ectoine/5-hydroxyectoine catabolic operon that is expressed from a substrate-inducible and EnuR regulated promoter positioned upstream of the first gene in the operon, *uehA* (Schulz et al., 2017b). The gene cluster encodes an ectoine/5-hydroxyectoine specific binding-protein-dependent TRAP transporter (UehABC) (Lecher et al., 2009), genes for enzymes hydrolyzing the ectoine and 5-hydroxyectoine ring to form α-ADABA/hydroxy-α-ADABA (EutD), to deacetylate these metabolites forming DABA and hydroxy-DABA (EutE), and enzymes (Ssd, Atf) mediating the catabolism of DABA to L-aspartate (Mais et al., 2020). The metabolism of 5-hydroxyectoine requires the function of the EutABC enzymes for its use as a nutrient by *R. pomeroyi* DSS-3 (Schulz et al., 2017b) but their precise enzymatic activities are unknown. The catabolic ectoine/5-hydroxyectoine gene cluster also contains two putative regulatory genes with experimentally untested functions: *usp* encodes a member of the universal stress proteins (Schweikhard et al., 2010), and *asnC* [also referred to as *deoX* (Schwibbert et al., 2011)] encodes a member of the feast and famine regulatory proteins (Yokoyama et al., 2006). **(B)** Biochemistry of the catalytic core of the ectoine/5-hydroxyectoine degradation route (Mais et al., 2020) as described in (A) and in the main text. The precise steps catalyzed by the EutABC enzymes in the catabolism of 5-hydroxyectoine (Schulz et al., 2017a) are uncertain. 2-OG: 2-oxoglutarate.

Ectoine nutrient utilization regulator (EnuR) [also referred to as EhuR (Yu et al., 2017), or EutR (Suvorova and Rodionov, 2016)] is a member of the MocR/GabR family (Suvorova and Rodionov, 2016; Pascarella, 2019) and serves as a repressor protein involved in the transcriptional control of ectoine/5-hydroxyectoine catabolic gene clusters (Jebbar et al., 2005; Schulz et al., 2017a,b; Figure 1A). The tetrahydropyrimidines ectoine and 5-hydroxyectoine (Galinski et al., 1985; Inbar and Lapidot, 1988) are among the most widely synthesized compatible solutes by members of the *Bacteria* (da Costa et al., 1998; Pastor et al., 2010; Czech et al., 2018; Hermann et al., 2020; Imhoff et al., 2020). Their accumulation is used by microorganisms to fend off the detrimental consequences of high osmolarity on cellular hydration and extremes in temperatures on growth (Pastor et al., 2010; Czech et al., 2018; Kunte et al., 2020).

The nitrogen-rich ectoines can also be used by microorganisms as nutrients (Galinski and Herzog, 1990; Onraedt et al., 2004; Jebbar et al., 2005; Vargas et al., 2006; Schwibbert et al., 2011; Landa et al., 2017; Schulz et al., 2017b; Reshetnikov et al., 2020; Nowinski and Moran, 2021). As ectoines are present in the environment in low concentrations (Mosier et al., 2013; Warren, 2014; Bouskill et al., 2016), their uptake requires high-affinity transport systems (Grammann et al., 2002; Jebbar et al., 2005; Hanekop et al., 2007; Kuhlmann et al., 2008; Lecher et al., 2009; Kuhlmann et al., 2011). Once imported, the tetrahydropyrimidine rings of ectoine and 5-hydroxyectoine are opened by the ectoine/5-hydroxyectoine hydrolase EutD (EC 3.5.4.44) to form α-acetyldiaminobutyric acid (α-ADABA) from ectoine and hydroxy-α-acetyldiaminobutyric acid (hydroxy-α-ADABA) from 5-hydroxyectoine. These metabolites are then further processed by the *N*-acetyl-L-2,4-diaminobutyric acid deacetylase EutE (EC 3.5.1.125) to diaminobutyric acid (DABA) in the case of ectoine, and possibly to hydroxy-DABA in the case of 5-hydroxyectoine (Figure 1B; Schwibbert et al., 2011; Mais et al., 2020). The presence of both EutD and EutE is needed to efficiently degrade ectoine and 5-hydroxyectoine. In this enzyme bimodule, EutE presumably supports the release of α-ADABA from the EutD active site through transient interactions (Mais et al., 2020). Eventually, the metabolites generated through the joint EutD/EutE enzyme activities are converted by additional enzymes encoded in ectoine/5-hydroxyectoine catabolic gene clusters, or elsewhere in the genome sequence, to L-aspartate to fuel the TCA-cycle (Schwibbert et al., 2011; Hermann et al., 2020; Mais et al., 2020). It should be noted in this context that the gene content of ectoine/5-hydroxyectoine catabolic clusters are variable (Schwibbert et al., 2011; Schulz et al., 2017b; Hermann et al., 2020; Reshetnikov et al., 2020), and consequently, the complete degradation route(s) of ectoines are not fully understood.

In contrast to the expression of ectoine/5-hydroxyectoine biosynthetic gene clusters that are typically induced in response to osmotic stress, the transcription of those for ectoine/5-hydroxyectoine catabolic operons is substrate inducible (Jebbar et al., 2005; Schulz et al., 2017b; Yu et al., 2017). However, externally provided ectoines are not the true inducers. Instead, the ectoine-derived metabolites α-ADABA and DABA serve this function by reacting with the PLP molecule covalently attached to the ATD of EnuR (Schulz et al., 2017a,b; Yu et al., 2017). Ectoine/5-hydroxyectoine importer and catabolic gene clusters are often juxtapositioned to a gene (*enuR*) encoding EnuR-type proteins (Schulz et al., 2017a; Hermann et al., 2020). This genetic arrangement implies a wider role for the EnuR repressor in controlling the use of ectoines as nutrients.

We use the marine α-proteobacterium *Ruegeria pomeroyi* DSS-3, a member of the widely distributed and ecophysiologically important *Roseobacter* clade (Moran et al., 2004), as our model system for the analysis of the catabolism of ectoines (Schulz et al., 2017a,b; Hermann et al., 2020; Mais et al., 2020). In contrast to other members of the *Roseobacter* clade (Simon et al., 2017), *R. pomeroyi* DSS-3 cannot synthesize ectoines (Schulz et al., 2017b). The ectoine/5-hydroxyectoine importer and catabolic operon of *R. pomeroyi* DSS-3 comprises 12 genes that are transcribed as a 12 kbp poly-cistronic mRNA (Figure 1A). The substrate-mediated induction of the transcription of this operon is carried out by a predicted sigma-70-type promoter, while the expression of the *enuR* gene, juxtapositioned to the importer and catabolic genes, occurs constitutively at a low level (Schulz et al., 2017b).

The external supply of 5-hydroxyectoine triggers a substantially stronger induction of expression of the ectoine/5-hydroxyectoine catabolic gene cluster in comparison with that afforded by ectoine (about 4-fold) (Schulz et al., 2017a). Binding of ectoine-derived α-ADABA to the purified EnuR protein has been demonstrated (*K*_d_ of about 1.7 μM) (Schulz et al., 2017a) but it is unclear if the initial hydrolysis product of 5-hydroxyectoine, hydroxy-α-ADABA (Mais et al., 2020), will also interact with EnuR to serve as a metabolically derived internal inducer. Here, we address this question through biochemical and bioinformatical analysis of the EnuR protein from *R. pomeroyi* DSS-3. We show through mass spectrometry that the ATD of EnuR contains a covalently bound PLP and that the initial hydrolysis products of ectoine and 5-hydroxyectoine, α-ADABA and hydroxy-α-ADABA, can be found in cells catabolizing ectoines. These metabolites serve as high-affinity ligands for EnuR with dissociation constants (*K*_d_) in the low micromolar range. We used molecular modelling and docking experiments to provide a view into the cofactor and inducer binding site of this transcription factor. Putative EnuR binding sites overlap core elements of the substrate-inducible sigma-70 type promoter of the catabolic gene cluster and the EnuR repressor protein binds to a 48 bp DNA fragment containing the corresponding promoter/regulatory region with a *K*_d_-value of about 2 μM.

## Results

### Purification and Biochemical Assessment of Ectoine Nutrient Utilization Regulator and Its Separate Aminotransferase Domain

**FIGURE 2:**
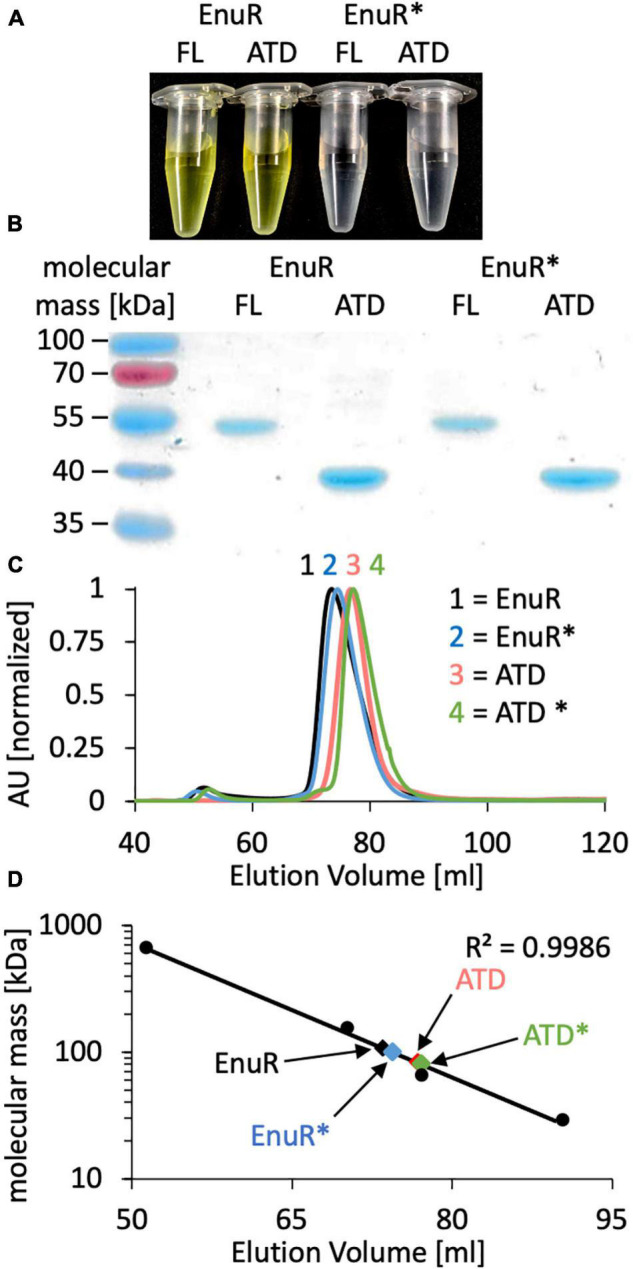
Biochemical analysis of EnuR. **(A)** The full-length (FL) wild-type EnuR protein from *R. pomeroyi* DSS-3, the EnuR^∗^ mutant (Lys302His) derivative unable to bind PLP (Schulz et al., 2017a) and the corresponding ATDs from both proteins were purified via affinity chromatography. The protein concentration in each sample was set to 50 μM and the yellow color of samples indicates the presence of a covalently bound PLP molecule. **(B)** The purity of the isolated proteins was analyzed by SDS-PAGE (12% polyacrylamide). Proteins were visualized by staining the gel with Instant*Blue*. **(C)** Analysis of the quaternary assembly of the full-length and ATD proteins of the wild-type EnuR protein and its EnuR^∗^-mutant derivative by size exclusion chromatography (SEC) on a HiLoad 16/600 Superdex 200 pg column. The positions at which these protein species eluted from the SEC-column were as follows: EnuR = 73.50 ml; EnuR^∗^ = 74.42 ml; ATD = 76.75 ml, and ATD^∗^ = 77.12 ml. AU: absorbance unit at a wavelength of 280 nm. **(D)** Comparison of the elution volumes of EnuR and ATD-proteins and their respective Lys302His derivatives with respect to the chromatographic behavior of marker proteins (thyroglobulin, 669 kDa; alcohol dehydrogenase, 150 kDa; bovine albumin, 66 kDa; carbonic anhydrase, 29 kDa). From this chromatographic behavior the following molecular masses were estimated: EnuR = 109 kDa; ATD = 83 kDa; EnuR^∗^ = 101 kDa and ATD^∗^ = 81 kDa, indicating that each of these proteins forms dimers in solution. The calculated molecular masses of the studied proteins fused to a *Strep*-tag II affinity peptide are: EnuR = 104 kDa; ATD = 80 Da; EnuR^∗^ = 104 kDa, and of the ATD^∗^ = 80 kDa.

To biochemically characterize EnuR from *R. pomeroyi* DSS-3 further, we expressed a full-length recombinant EnuR-*Step*-Tag-II protein heterologously in *E. coli* and purified it to apparent homogeneity via affinity chromatography on a Streptactin column. We also separately expressed and purified the C-terminal ATD of the wild-type protein as a *Strep*-Tag-II fusion protein. In addition, we carried out similar types of production and affinity purification experiments with a variant of the *R. pomeroyi* DSS-3 EnuR protein in which the Lys residue to which the PLP cofactor is presumably covalently attached is replaced by a His residue (EnuR^∗^; Lys302His). As a result of this amino acid substitution, PLP cannot be covalently bound, leading to the loss of the characteristic PLP-dependent yellow color of the full-length wild-type EnuR protein solutions (Schulz et al., 2017b; Figures 2A,B). Incorporation of the PLP cofactor also occurred during the heterologous production of the ATD from the wild-type protein but not into the ATD derived from the EnuR^∗^ protein (Figure 2A).

The GabR protein from *B. subtilis* is a homodimer where the monomers are arranged in a head to tail configuration (Edayathumangalam et al., 2013) and where the isolated ATD can form homodimers as well (Okuda et al., 2015a). In a similar vein, we found that the purified EnuR protein and its separately produced ATD also assemble into homo-dimers in solution. These proteins eluted from a size exclusion column as corresponding to 109 kDa and 83 kDa molecular mass species, respectively (Figures 2C,D). The calculated molecular mass of the *R. pomeroyi* DSS-3 monomeric EnuR protein is 51 kDa, while that of its ATD is 40 kDa. The EnuR^∗^ protein and its isolated ATD^∗^ behaved in these chromatography experiments identical to that of the corresponding wild-type proteins (Figures 2C,D). Taken together, these biochemical experiments show that (i) dimer-formation of EnuR depends on its ATD, regardless whether the ATD carries a covalently attached PLP or not, and (ii) that the PLP cofactor can be attached to the ATD even when the N-terminal DNA-reading head of EnuR is missing.

### Verification of Bound Pyridoxal-5′-Phosphate in Ectoine Nutrient Utilization Regulator and Its ATD by Mass Spectrometry

**FIGURE 3:**
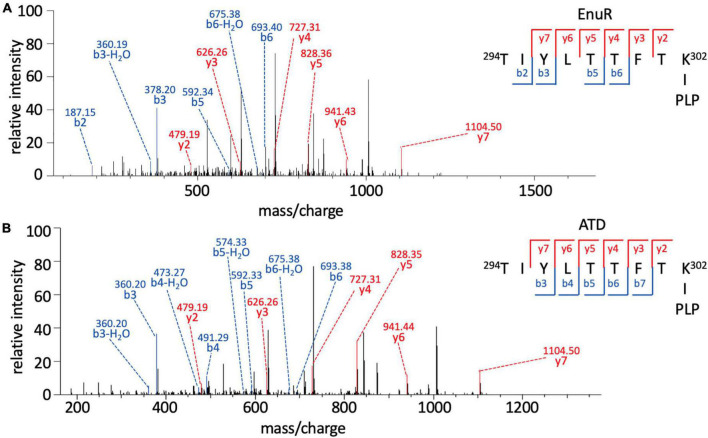
Pyridoxal-5′-phosphate is covalently bound to Lys302 (K302) of EnuR. MS/MS spectrum of the PLP-containing peptide spanning residues 294-302 (TIYLTTFTK∼PLP) of **(A)** full-length EnuR and **(B)** EnuR-ATD. EnuR and EnuR-ATD were reduced with NaBH_4_ before tryptic digestion of the proteins to stabilize the otherwise acid-labile aldimine. These tryptic digests were subjected to analysis by mass spectrometry. The peptide ^294^TIYLTTFTK^302^ exhibited a mass-to-charge ratio (m/z) of 1,318.63305 (M + H^+^ ion), although its theoretical mass-to-charge ratio is only 1,087.60339 (M + H^+^ ion); the difference of 231.02966 is in good agreement with the mass of a PLP. To narrow down the position of PLP attachment in the ^294^TIYLTTFTK^302^ peptide ion, this precursor ion was further fragmented by MS/MS resulting in breaks of the precursor ion at the peptide bonds, giving rise to b-ions (*N*-terminal ion, blue) and y-ions (C-terminal, red), respectively. The detected b-ions correspond to the theoretical masses of the unmodified amino acids contained therein; however, all y-ions show the difference in mass corresponding to PLP, e.g., the y2 ion has a theoretical mass of 248.16187 but exhibits 479.19387 experimentally in this MS/MS (difference of 231.03200). As residue Thr301 is unable to covalently bind PLP, this consolidates amino acid Lys302 of EnuR as the PLP-binding residue.

In order to probe whether the PLP co-purifying with EnuR is covalently attached to Lys302 or just coordinated by this residue, we subjected the EnuR and EnuR-ATD proteins to mass spectrometric (MS) analysis. Due to the lability of the aldimine group that would be formed between the ε-amino group of the lysine side chain and the PLP cofactor, the purified proteins were reduced with NaBH_4_ prior to tryptic digestion. In the subsequent MS analysis, we retrieved peptides spanning amino acid residues 294-302 of both the EnuR and EnuR-ATD proteins that exhibited a mass-to-charge (m/z) ratio of 1,318.63305 (theoretical mass-to-charge ratio of M + H^+^ of 1,087.60339), corresponding to a mass shift of 231.02966 Da, a value in excellent agreement with the presence of a covalently attached PLP (theoretical difference of 231.029662 Da). Further MS/MS fragmentation of these peptides corroborates that Lys302 is the site of attachment for PLP (Figures 3A,B). This consolidates the previous suggestion that EnuR binds PLP, similar to type I aminotransferases and MocR/GabR-type regulators (Tramonti et al., 2018), through the formation of an aldimine between an evolutionary conserved lysine residue (Lys302 in EnuR) and the cofactor (Schulz et al., 2017a).

### Inducer-Binding by the Purified Ectoine Nutrient Utilization Regulator Protein and Its Isolated ATD

**FIGURE 4:**
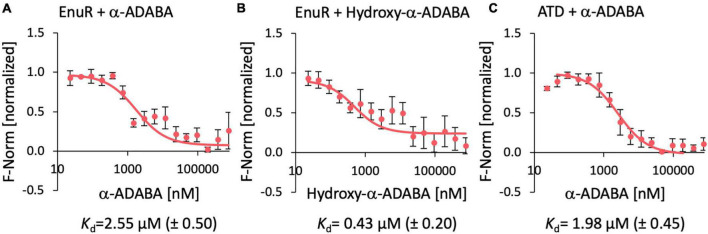
Ligand binding by the wild-type EnuR protein and its isolated ATD as assessed by microscale thermophoresis. Purified EnuR protein (200 nM) was incubated with increasing concentrations of α-ADABA **(A)**, Hydroxy-α-ADABA **(B)**, and the C-terminal ATD of EnuR was incubated with α-ADABA **(C)**. The ability of these compounds to bind to the EnuR protein **(A,B)** or its isolated ATD **(C)** was measured by microscale thermophoresis. The ligands were titrated to a constant amount (200 nM) of purified and PLP-containing EnuR protein, or its PLP-containing ATD. Error bars show the standard deviation; the ligand-binding experiments were repeated six times with independently purified and fluorescently labeled EnuR/ATD preparations. F-norm refers to the thermophoretic behavior of these tested proteins in a ligand gradient.

Micro-scale thermophoresis is a sensitive method that traces the movement of fluorescently labelled proteins in a temperature gradient in response to a ligand (Wienken et al., 2010). We used this method to determine the dissociation constant (*K*_d_) (Figure 4) for the binding of the known ectoine-derived inducer α-ADABA (Schulz et al., 2017a) and that of the presumed 5-hydroxyectoine-derived inducer hydroxy-α-ADABA (Figure 1B; Mais et al., 2020). Both compounds were obtained via chemical synthesis through alkaline-mediated hydrolysis of the tetrahydropyrimidine ring of either ectoine (Kunte et al., 1993) or 5-hydroxyectoine. They were purified by repeated chromatography on silica columns to apparent homogeneity as assessed by NMR-spectroscopy (Mais et al., 2020).

Ectoine nutrient utilization regulator bound α-ADABA with a *K*_d_ of 2.55 ± 0.5 μM (Figure 4A), a value that fits well with a previous measured *K*_d_-value of 1.7 ± 0.3 μM for this ligand (Schulz et al., 2017a). The full length EnuR protein exhibited a *K*_d_-value of 0.43 ± 0.2 μM for hydroxy-α-ADABA (Figure 4B). We also assessed the binding of α-ADABA to the PLP-bound ATD of the wild-type EnuR protein in a micro-scale thermophoresis ligand-binding experiment. We found that this domain bound this ectoine metabolite with approximately the same *K*_d_-value (1.98 ± 0.45 μM) (Figure 4C) as the full length EnuR protein (*K*_d_ of 2.55 ± 0.5 μM).

### Modeling the Overall Fold of Ectoine Nutrient Utilization Regulator and Phylogenomic Conservation of This Repressor Protein

**FIGURE 5:**
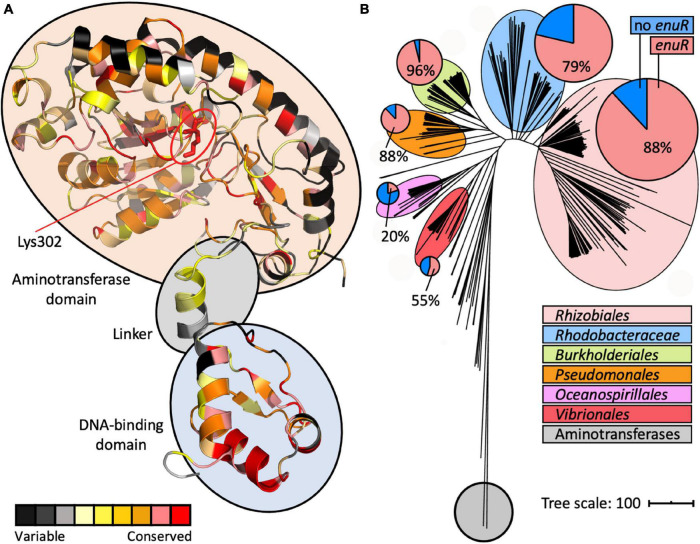
A model of the structure of a EnuR monomer and phylogenomic distribution of *enuR*-type genes in bacteria encoding EutD/EutE ectoine/5-hydroxyectoine degrading enzymes. **(A)** A model of the *R. pomeroyi* DSS-3 EnuR protein was established using the Phyre^2^ protein structure prediction server (Kelley et al., 2015) that is based on the crystal structure of the *B. subtilis* GabR protein (Edayathumangalam et al., 2013). Each amino acid in this model is color coded by its degree of conservation in EnuR-type proteins as assessed with the ConSurf algorithm (Berezin et al., 2004). An amino acid sequence alignment of 278 EnuR-type proteins was used to calculate the conservation at each position of the protein chain. The predicted domain-organization of EnuR with its N-terminal DNA-reading head containing a winged-helix-turn-helix motif, the flexible linker region, and the C-terminal ATD containing the Lys residue (Lys302) to which the PLP cofactor is covalently attached, is indicated. **(B)** iTol (Letunic and Bork, 2019) generated tree of the ectoine/5-hydroxyectoine hydrolase EutD representing 363 EutD-type proteins (Mais et al., 2020). The EutD protein-based tree was rooted with *E. coli* and *Homo sapiens* aminopeptidases (Bradshaw et al., 1998; Wilce et al., 1998). All microorganisms possessing EutD-type ectoine/5-hydroxyectoine hydrolases were analyzed for the presence of *enuR*-type genes juxtapositioned to ectoine/5-hydroxyectoine catabolic gene clusters; larger phylogenetic groups possessing such genetic arrangements are highlighted. In the circles, the red segments represent the number of microorganisms within a given phylogenetic group that possess *enuR*-type genes, while the blue segment represents the number of those bacteria that lack it.

Since we wanted to further understand the molecular determinants for inducer binding by EnuR, we generated a structural model of its monomer. The EnuR model was fashioned on the crystal structure of the *B. subtilis* GabR protein (Edayathumangalam et al., 2013), the only GabR/MocR-type regulatory protein whose crystal structure is known. Our EnuR model (Figure 5A) was generated using Phyre^2^ (Kelley et al., 2015) set in the extensive mode^1^. An overlay of the GabR protein and of the EnuR model revealed a root mean square deviation (RMSD) of just 0.86 Å (over 390 amino acids), indicating that the overall fold of these two MocR/GabR-type regulatory proteins is most likely closely related.

In addition to the modelling of the putative EnuR structure, we also assessed the phylogenomic conservation of this regulatory protein. For this analysis, we relied on a recently reported manually curated dataset assessing the presence of ectoine/5-hydroxyectoine catabolic gene clusters in 8 850 microbial genome sequences (Mais et al., 2020). The identification of putative ectoine/5-hydroxyectoine catabolic gene clusters was based upon a direct juxtaposition of the *eutD* and *eutE* genes. 363 microbial genome sequences contained *eutD/eutE* pairs. We found that 76% (278 out of 363) of the corresponding ectoine/5-hydroxyectoine catabolic gene clusters contained a juxtapositioned *enuR* gene (Figure 5B). We then used the information gleaned from this bioinformatic approach (Supplementary Figure 1) to assess the degree of amino acid conservation at each position in the EnuR protein chain (Supplementary Figure 2). Subsequently, we projected these data onto our EnuR model by using the ConSurf algorithm (Berezin et al., 2004; Figure 5A).

In an alignment of the 278 retrieved EnuR-type proteins, we found that the degree of amino acid sequence identity ranged between 82% (for the EnuR protein from *Leisingeria* sp. NJS201) and 40% (for the EnuR protein from *Salipiger pacificus* YSBP01) when the *R. pomeroyi* DSS-3 EnuR protein was used as the search query. As expected, those amino acid residues forming the N-terminally positioned winged-helix-turn-helix DNA reading head are particularly well conserved, as are central segments of the ATD (Figure 5A and Supplementary Figure 2). Notably, the Lys residue in the ATD to which the PLP molecule is covalently attached in EnuR (Lys302 in the *R. pomeroyi* DSS3 EnuR protein) (Schulz et al., 2017b), is completely conserved among the 278 inspected EnuR-type proteins (Supplementary Figure 2).

Among those 363 genome sequences that contain *eutD/eutE* pairs, six major microbial orders are represented (Figure 5B), all of which belong to the proteobacteria (Supplementary Table 1). Using computational tools provided via the IMG/M web-server (Chen et al., 2021), a phylogenetic tree was calculated for the 363 EutD-type proteins using aminotransferases from *Escherichia coli* (Wilce et al., 1998) and *Homo sapiens* (Bradshaw et al., 1998) as outgroups. The EutD-derived tree was visualized using the iTOL software suit (Letunic and Bork, 2019), and the presence of 278 *enuR*-type genes was then projected onto this tree (Figure 5B; Supplementary Figure 1). *enuR*-type genes are dominantly represented among *Rhizobiales, Rhodobacterales, Burkholderiales, Pseudomonales, Oceanospirillales*, and *Vibrionales* predicted to use ectoines as nutrients (Figure 5B).

### Molecular Docking of α-ADABA, hydroxy-α-ADABA, γ-ADABA, and DABA Into the ATD of Ectoine Nutrient Utilization Regulator Reveals the Likely Molecular Determinants for Inducer-Binding

**FIGURE 6:**
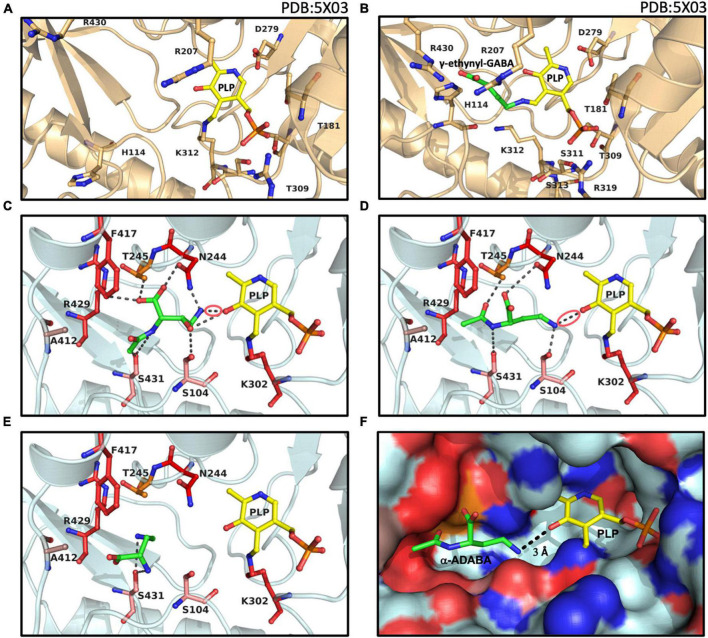
Structural views into the presumed inducer binding sites of GabR and EnuR. The crystal structure of the dimeric *B. subtilis* GabR protein (PDB accession code: 5XO3) (Edayathumangalam et al., 2013) contains **(A)** in one monomer a PLP molecule covalently attached to a the side-chain of Lys312 and in the second monomer **(B)** a PLP molecule ligated to γ-ethynyl-GABA (a mimic of GABA). Docking models of the ectoine/5-hydroxyectoine derived inducers (green) **(C)** hydroxy-α-ADABA, **(D)** α-ADABA, and **(E)** DABA docked into the presumed EnuR ligand binding cavity. The ligands and EnuR amino acid residues predicted to be involved in inducer-binding are displayed as sticks. The protein backbone is shown in gold for the GabR protein **(A,B)**, cyan for the EnuR protein **(C,D,E)**, and the PLP cofactor covalently attached to the side-chain of Lys302 in EnuR is depicted in yellow. The aldimine bonds formed by the chemical interaction of the inducers and PLP in the process of the external aldimine formation (Tramonti et al., 2018) are indicated by red circles in **(C,D)**. **(F)** Surface representation of the EnuR ligand and PLP cofactor binding cavity with the predicted position of the α-ADABA molecule. Positively and negatively charged segments of the binding cavity are labeled in blue and red, respectively. The graphical representation of the ligand binding sites in each of the shown panels was rendered with PyMol (Delano, 2002).

The crystal structure of the dimeric full-length *B. subtilis* GabR protein contained in the cofactor and inducer binding site of one of its monomers a PLP molecule covalently bound to a Lys residue (Figure 6A). In the second monomer, a free PLP molecule was found that was chemically ligated to γ-ethynyl-GABA, a substrate-mimic of GABA (Figure 6B), thereby revealing the structure of the external aldimine (Edayathumangalam et al., 2013). In addition, a crystal structure of the isolated dimeric ATD of GabR captured the GABA-mediated structural transition catalyzed by the conversion of the internal aldimine to the external aldimine (Park et al., 2017). Collectively, these crystal structures guided our docking experiments of the high affinity inducers α-ADABA and hydroxy-α-ADABA into the presumed ligand-binding site of EnuR. The docking experiments were carried out using AutoDock Vina (Trott and Olson, 2010).

In our EnuR model, hydroxy-α-ADABA is bound in close proximity to the PLP molecule with which it interacts via its free amino group. Nine interactions of the inducer molecule with the EnuR protein are observed (Figure 6C). The oxygen of PLP interacts with the hydroxyl-oxygen of hydroxy-α-ADABA as well as with the primary amino group of this molecule. This nitrogen atom is also bound by the O-1 atom of Asn244. Additionally, hydroxy-α-ADABA is stabilized by interactions with the N-2 atom of Asn244 and through interactions with Thr245, Phe417, Ser431, Ser104 (Figure 6C). Collectively, these nine interactions are the foundation for the high affinity of EnuR for hydroxy-α-ADABA (Figure 4B); they thereby establish the orientation of this 5-hydroxyectoine-derived metabolite in the inducer binding site of EnuR. A more detailed description of the energetics of these interactions are summarized in Supplementary Table 2.

Compared to hydroxy-α-ADABA, α-ADABA appears to be a slightly more linear molecule due to the lack of the hydroxy group. Consequently, it is positioned in the predicted inducer binding site of EnuR in a slightly different orientation (Figure 6D). Like hydroxy-α-ADABA, the primary amino group of α-ADABA interacts with the oxygen atom of PLP. A second interaction of the primary amino group of α-ADABA is found with the side chain of Ser104, a configuration different from that predicted for hydroxy-α-ADABA (Figures 6C,D). As observed for hydroxy-α-ADABA, the carboxyl group of α-ADABA interacts with the nitrogen atom present in the side chain of Asn244. The secondary nitrogen atom of α-ADABA interacts with the oxygen atom of Ser431. In total five interactions of EnuR with α-ADABA are predicted (Figure 6D and Supplementary Table 2), suggesting that this compound will be bound with a somewhat lower affinity by EnuR in comparison with hydroxy-α-ADABA. This is precisely what we observed in our *in vitro* ligand binding experiments where the affinity of EnuR for hydroxy-α-ADABA was about five times higher than that for α-ADABA (*K*_d_ of about 0.43 μM versus 2.55 μM for hydroxy-α-ADABA and α-ADABA, respectively) (Figures 4A,B).

The amino acid sequence alignment of 278 EnuR-type proteins revealed a high degree of conservation of the amino acids predicted to be involved in hydroxy-α-ADABA and α-ADABA binding by our docking studies (Figures 6C,D). Especially Ser104, Asn244, Lys302, Phe417, and Arg429 are either strictly or highly conserved (Supplementary Table 3). Slight deviations can be observed for the position of Ser431; however, this amino acid is mainly exchanged to a Cys residue (135/278). In our model of the EnuR ligand binding cavity, the sulfur atom of Ser431 interacts directly with both inducer molecules and it is thus a reasonable assumption that the sulfur atom of the Cys side chain will adopt the same interaction. The positions Ala412 and Thr245, more peripheral residues in the ligand binding site (Figures 6C,D), seem to be less important for the binding of the hydroxy-α-ADABA and α-ADABA molecules. Ala412 is frequently substituted by a Leu residue (123/278), while a great variety of residues can assume the position of Thr245 in EnuR-type proteins (Supplementary Table 3). A visualization of the EnuR binding site for the PLP cofactor and the inducer α-ADABA is rendered in Figure 6F.

The ectoine metabolite diaminobutyric acid (DABA) (Figure 1B) also serves as an inducer for EnuR (Schulz et al., 2017a; Yu et al., 2017). A *K*_d_-value of about 460 μM has been reported for the EnuR protein from *R. pomeroyi* DSS-3 (Schulz et al., 2017a). Hence, there is a substantial difference in affinity between DABA on one hand and α-ADABA and hydroxy-α-ADABA on the other hand for EnuR (Figures 4A,B). As a consequence of the low binding affinity of DABA for EnuR, we observed multiple positions for the DABA molecule within the presumed inducer binding site of EnuR in the first round of docking experiments. Optimization and refinement of these positions was difficult and only two interactions of DABA were observed that hinted at a possible binding state (Supplementary Table 2). However, in contrast to α-ADABA and hydroxy-α-ADABA (Figures 6C,D), this would position DABA too far away from the PLP molecule (Figure 6E; Tramonti et al., 2018) in order to serve its function as an inducer for EnuR. While the distances between the primary nitrogen group of α-ADABA and hydroxy-α-ADABA to the PLP cofactor in our EnuR model are 2.6 Å and 3 Å, respectively, the distance of the corresponding nitrogen group of DABA is about 7 Å. Consequently, the actual position of the low-affinity EnuR ligand DABA cannot be reliably predicted by our docking experiments.

The isomer of α-ADABA, γ-ADABA, is the substrate for the ectoine synthase EctC (Czech et al., 2019), the key enzyme for the production of ectoine (Peters et al., 1990; Ono et al., 1999; Hermann et al., 2020). We wondered if interactions of γ-ADABA with EnuR could be found in docking experiments but no stable binding was observed. This was mainly due to steric clashes with amino acid residues whose side chains protrude into the inducer binding site of EnuR. This result is fully consistent with previous experiments in which no binding of γ-ADABA to purified EnuR from *R. pomeroyi* DSS-3 could be measured (Schulz et al., 2017a). The failure to dock the non-inducer γ-ADABA into the EnuR binding site can be considered as an internal control for the successful docking experiments with its isomer α-ADABA, a high-affinity ligand for EnuR (Figure 4A).

### Targeted Metabolic Analysis of Ectoine- and 5-Hydroxyectoine-Derived Metabolites

**FIGURE 7:**
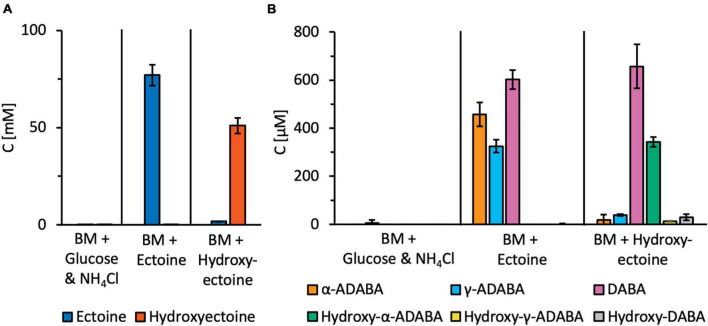
Targeted metabolic analysis of ectoine and 5-hydroxyectoine-derived metabolites. *R. Pomeroyi* DSS-3 cultures were grown in basal medium (BM) either in the presence of glucose and NH_4_Cl, or ectoines as sole carbon, energy, and nitrogen sources. Cultures were grown to an OD_578_ of about one, centrifuged, and the cells were washed once in BM. Subsequently, metabolites were extracted with 20% ethanol. Ectoine and 5-hydroxyectoine-derived metabolites were analyzed by HPLC-ESI-MS. For these experiments, four independent cultures were grown and a sample prepared from each of them was assayed twice. The indicated error bars show the standard deviation. **(A)** Presumed intracellular pools of the growth substrates ectoine and 5-hydroxyectoine, and **(B)** intracellular pools of ectoine and 5-hydroxyectoine derived metabolites.

Since α-ADABA, hydroxy-α-ADABA, DABA, and possibly also hydroxy-DABA can interact with EnuR and serve as inducers, we wondered if these compounds can be found in cells of *R. pomeroyi* DSS-3 actively catabolizing ectoines. We therefore performed targeted metabolic analysis of ectoine- and 5-hydroxyectoine-derived metabolites in cells that were grown with either ectoine or 5-hydroxyectoine as sole carbon, energy and nitrogen sources. The metabolic profile of these cultures was compared with that of cells using glucose as carbon and energy source and NH_4_Cl as nitrogen source in a chemically fully defined minimal medium. The analyzed samples contained substantial amounts of either ectoine or 5-hydroxyectoine, but these values represent in all likelihood not only intracellular pools of these compounds but probably also reflect incomplete removal during the harvesting and washing of the cells (Figure 7A).

Substantial amounts of α-ADABA were found in the extracts of cells grown in the presence of ectoine, while hydroxy-α-ADABA was found in cells grown in the presence of 5-hydroxyectoine (Figure 7B). Interestingly, DABA was found under both cultivation conditions, regardless whether the cells were grown in the presence of ectoine or of 5-hydroxyectoine. The pool of hydroxy-DABA in cells that received 5-hydroxyectoine as their sole carbon and nitrogen sources was very low and, as expected, not detectable in cells that were exposed to ectoine (Figure 7B). A rather surprising finding was the detection of substantial amounts of γ-ADABA in cells that were grown in the presence of ectoine, while γ-ADABA was present only in very low amounts in cells grown in the presence of 5-hydroxyectoine (Figure 7B).

### Binding of Ectoine Nutrient Utilization Regulator to the Promoter Region

Using comparative genomics and metabolic reconstruction, Suvorova and Rodionov (2016) have previously analyzed putative EnuR (EutR) operator binding sites in 69 microbial genomes. This analysis suggested a consensus operator sequence for EnuR (EutR)-type proteins that consists of an inverted repeat of five base pairs separated by six base pairs [ATTGTnnnnnnACAAT] (Suvorova and Rodionov, 2016). However, depending on the microbial species under study, variations on this theme exist (Schulz et al., 2017a; Yu et al., 2017).

**FIGURE 8:**
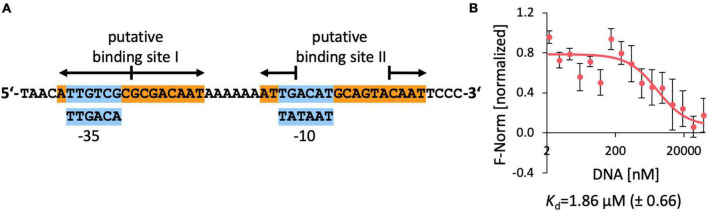
DNA-binding of EnuR to the promoter and regulatory region of the ectoine/5-hydroxyectoine catabolic gene cluster of *R. pomeroyi* DSS-3. **(A)** A 48 bp DNA fragment was used for the DNA-binding assays. Putative –35 and –10 regions (in blue boxes) of a sigma-70-type promoter (Feklistov et al., 2014) are indicated and predicted EnuR binding sites (Suvorova and Rodionov, 2016; Schulz et al., 2017a) are marked. **(B)** DNA binding activity of the *R. pomeroyi* DSS-3 purified EnuR protein containing PLP to the promoter region was assessed by microscale thermophoresis. The double-stranded 48 bp DNA regulatory fragment was titrated to a constant amount (200 nM) of purified and PLP containing EnuR protein. Error bars shows the standard deviation; the DNA binding experiment was repeated six times with independently purified and fluorescently labeled EnuR preparations. F-norm refers to the thermophoretic behavior of the tested protein in a ligand (= DNA) gradient.

In *R. pomeroyi* DSS-3, two closely spaced potential EnuR binding sites in the intergenic region between the 3′-end of *enuR* and the beginning of the ectoine/5-hydroxyectoine catabolic operon (Figure 1A) can be observed. They overlap with core elements (−10 and −35 sequences separated by 17 bp) of the predicted sigma-70 type promoter (Feklistov et al., 2014; Figure 8A) for the ectoine/5-hydroxyectoine catabolic gene cluster. DNA band-shift assays have previously shown that EnuR can specifically bind *in vitro* to a 278 bp DNA fragment carrying both predicted operators (Schulz et al., 2017a). The putative binding site one is a perfect repeat with eight bp in each of the half-sites, while the second putative binding site has an overall length of 18 bp and only the outermost four bp are a perfect inverted repeat (Figure 8A). It is currently unknown if both of the *in silico* predicted EnuR binding sites (Suvorova and Rodionov, 2016) are biological relevant to control the ectoine/5-hydroxyectoine importer and catabolic gene cluster from *R. pomeroyi* DSS-3.

Since no quantitative data for the interaction of the *R. pomeroyi* DSS-3 EnuR protein with its presumed operator(s) have been reported, we carried out DNA-binding assays with the purified full-length and PLP-containing EnuR protein with a labeled 48 bp DNA fragment containing both presumed EnuR binding sites using microscale thermophoresis. We measured a *K*_d_-value of 1.9 μM (Figure 8B). This is a higher yet physiologically relevant *K*_d_-value than the *K*_d_-value (9.14 nM) reported for the promoter/operator interaction of EnuR with its two established operators for the *S. meliloti* ectoine/5-hydroxyectoine catabolic gene cluster (Yu et al., 2017).

## Discussion

A computational classification of MocR/GabR-type regulators has previously shown that EnuR/EhuR-type proteins form a clade well separated from other sub-groups of MocR/GabR-type transcription factors (Pascarella, 2019). As studied in detail for the *B. subtilis* GabR protein, the PLP-dependent chemistry driving the transition of an internal to an external aldimine, and hence the ensuing interconversion of the DNA-binding status of MocR/GabR-type regulators, requires the formation of an aldimine bond between a previously covalently bound PLP with a primary nitrogen group present in a system-specific inducer molecule (e.g., GABA) (Edayathumangalam et al., 2013; Okuda et al., 2015a,b; Park et al., 2017; Wu et al., 2017; Tramonti et al., 2018). Spectroscopic and mutagenesis studies have previously suggested that the EnuR repressor from *R. pomeroyi* DSS-3 contains PLP as a cofactor that is covalently attached to the side chain of Lys302 (Schulz et al., 2017a). The mass-spectrometry data reported here (Figure 3) corroborate these previous reports. The presence of the Lys302-bound PLP cofactor is crucial for the induction process, as a mutant (Lys302His) (EnuR^∗^) unable to incorporate PLP into EnuR functions as a constitutive repressor leading to the inability of *R. pomeroyi* DSS3 to use ectoines as nutrients (Schulz et al., 2017a). The central role of this residue for the proper functioning of the EnuR repressor from *R. pomeroyi* DSS-3 is reflected in the strict conservation of the corresponding lysine residue in each of the 278 EnuR-type proteins that we have inspected (Figure 5A).

Despite being strong inducers of their catabolic importer and degradative gene cluster in *R. pomeroyi* DSS-3 (Schulz et al., 2017a,b), externally provided ectoines cannot directly serve such a regulatory function as these tetrahydropyrimidines lack a primary amino group that would allow them to chemically interact with the covalently bound PLP cofactor present in EnuR (Figure 1B). Such a primary amino group is, however, present in each of the initial hydrolysis products of ectoine and 5-hydroxyectoine, α-ADABA and hydroxy-α-ADABA, respectively, metabolites formed by the EutD enzyme, and in their deacetylated derivatives, DABA and hydroxy-DABA, respectively, formed by the EutE enzyme (Figure 1B; Schwibbert et al., 2011; Hermann et al., 2020; Mais et al., 2020). Collectively, our data suggest that these four metabolically derived compounds of ectoine/5-hydroxyectoine serve as internal inducers for the EnuR repressor. Although likely, we cannot be certain of such a role for hydroxy-DABA as this compound is currently not available in amounts required for ligand binding experiments.

α-ADABA and DABA have already been shown to serve as ligands for the *R. pomeroyi* DSS-3 EnuR protein (Schulz et al., 2017a). DABA has also been shown to serve such a role for the related protein from *S. meliloti*, although the affinity of the corresponding EnuR repressor for this ligand is unknown (Yu et al., 2017). We show here that hydroxy-α-ADABA interacts in a high affinity process with EnuR. EnuR bound hydroxy-α-ADABA with a *K*_d_-value of 0.43 ± 0.2 μM, an about five-fold improved affinity compared with α-ADABA (*K*_d_ of 2.55 ± 0.5 μM) (Figure 4). On the other hand, the *R. pomeroyi* DSS-3 EnuR protein binds DABA with a *K*_d_-value of about 460 μM (Schulz et al., 2017a). Hence, the affinities of EnuR for the primary, EutD-mediated hydrolysis products of ectoine and 5-hydroxyectoine, are about 180- and 1,000-fold higher than those for DABA.

The central ectoine/5-hydroxyectoine catabolic enzymes, the hydrolase EutD and the deacetylase EutE, operate as a bi-module in the sense that both enzymes have to be present to efficiently degrade ectoines (Supplementary Figure 3), although a stable EutD/EutE protein complex has yet to be observed *in vitro* (Mais et al., 2020). This raises the question whether the two high-affinity inducers of EnuR, α-ADABA and hydroxy-α-ADABA, are actually present in the cell and are not immediately deacetylated to produce the medium-affinity inducers DABA and hydroxy-DABA (Figure 1B). Through targeted metabolic analysis of cultures grown either in the presence of ectoine or 5-hydroxyectoine, we detected considerable amounts of α-ADABA and hydroxy-α-ADABA, respectively, in the cells (Figure 7B), so that these compounds will readily be able to interact with EnuR. Sizable amounts of the inducer DABA were also detected in cells grown in the presence of either ectoine or 5-hydroxyectoine, while hydroxy-DABA was found only in rather low concentrations and, as expected, only in cells grown on 5-hydroxyectoine (Figure 7B). The substantial amounts of DABA in the cytoplasm of cells of these latter cultures indicate that hydroxy-DABA is rapidly converted into DABA, a finding that needs to be taken into account for further studies on the enzymology of the ectoine/5-hydroxyectoine catabolic route (Figure 1B; Schwibbert et al., 2011; Hermann et al., 2020; Mais et al., 2020; Reshetnikov et al., 2020).

Our docking experiments with EnuR involving α-ADABA and hydroxy-α-ADABA as ligands (Figures 6C,D,F) do not capture the chemical interconversion of the internal to the external aldimine crucial for the change in the DNA-binding properties of MocR/GabR-type regulators (Edayathumangalam et al., 2013; Okuda et al., 2015b; Park et al., 2017; Wu et al., 2017; Tramonti et al., 2018). Despite this limitation, our *in silico* experiments should provide a solid structure-based view into the cofactor and inducer binding site of the EnuR repressor (Figures 6C,D,F). This conclusion is supported by the fact that our unconstrained docking experiments position the primary nitrogen group present in both inducers in close distance, 3 Å for hydroxy-α-ADABA and 2.6 Å for α-ADABA, to the PLP cofactor with whom they have to interact. These distances are well suited for a chemical reaction between the bound PLP and the inducers (Figures 6C,D,F). Most of the EnuR residues predicted by our modeling studies to interact with hydroxy-α-ADABA or α-ADABA are either completely or functionally conserved in the 278 EnuR-type proteins onto which our analysis relied (Figure 5A) (Supplementary Figure 1 and Supplementary Table 3).

The isomer of α-ADABA, γ-ADABA, is enzymatically generated during ectoine biosynthesis and serves as the substrate for the ectoine synthase EctC (Czech et al., 2019). It does not serve as an inducer for EnuR (Schulz et al., 2017a). Consistent with previous ligand-binding experiments, we were unable to place γ-ADABA into the presumed inducer-binding site of EnuR in our docking experiments. Assuming that the core of the γ-ADABA molecule would localize in the same manner in the EnuR ligand-binding cavity as its isomer α-ADABA so that its primary amino group would be positioned toward the PLP cofactor, then the carboxyl moiety of γ-ADABA would be placed in close proximity to the side chain of Asn244 (Figure 6D). This would lead, in all likelihood, to a sterical clash, and binding of γ-ADABA should thereby be prevented, or at least strongly disfavored (Figures 6D,F). Collectively, these are physiologically important findings as the central intermediate in ectoine synthesis, γ-ADABA, can consequently not trigger ectoine catabolism in those microorganisms capable to both synthesize and degrade ectoines (Schwibbert et al., 2011; Czech et al., 2018; Hermann et al., 2020; Mais et al., 2020).

The detection of γ-ADABA in cells grown in the presence of ectoine (Figure 7B) comes as a true surprise as *R. pomeroyi* DSS-3 cannot synthesize ectoine (Moran et al., 2004; Schulz et al., 2017b). Previous *in vivo* and *in vitro* experiments of recombinant EutD and EutE enzymes prepared in *E. coli* indicated that γ-ADABA did not play any role in the catabolism of ectoines by *R. pomeroyi* DSS-3 (Mais et al., 2020). However, in *H. elongata*, a bacterium that can both degrade and synthesize ectoine, γ-ADABA was an intermediate in ectoine catabolism, potentially re-used for ectoine biosynthesis (Schwibbert et al., 2011). This is not possible in the marine bacterium *R. pomeroyi* DSS-3. Our detection of γ-ADABA raises the question if this ectoine biosynthetic precursor is a dead-end by-product of ectoine catabolism in *R. pomeroyi* DSS-3, or alternatively, which physiological function it might serve. Notable, no γ-ADABA was detected in cells of halotolerant methylotrophs metabolizing ectoine (Reshetnikov et al., 2020).

The data reported here for hydroxy-α-ADABA, and those that were previously provided for the binding of α-ADABA and DABA to EnuR (Schulz et al., 2017a,b; Yu et al., 2017), lend themselves to a straight-forward regulatory model for the encounter of microbial ectoine/5-hydroxyectoine consumers with environmental ectoines (Mosier et al., 2013; Warren, 2014; Bouskill et al., 2016). In the absence of ectoines, the *R. pomeroyi* DSS-3 importer and catabolic gene cluster is expressed at a very low level (Schulz et al., 2017a). Nevertheless, this basal level of transcription is sufficient to allow import of trace amounts of ectoines via the high affinity TRAP-type UehABC uptake system from *R. pomeroyi* DSS-3 (Lecher et al., 2009). Subsequent to the initial import of ectoines, their catabolism will set in at a low level, thereby forming limited pools of the high-affinity EnuR inducers α-ADABA and hydroxy-α-ADABA, and the medium affinity inducers DABA (and potentially hydroxy-DABA as well). Their interactions with the covalently bound PLP cofactor will relieve EnuR-mediated repression of the transcriptional activity of the substrate-inducible promoter (Figures 1A, 8A). Consequently, enhanced and subsequently sustained increased expression of the ectoine/5-hydroxyectoine importer and catabolic gene cluster will ensue, thereby promoting increased import and catabolism of ectoines. As the inducers α-ADABA, hydroxy-α-ADABA, DABA and hydroxy-DABA are early intermediates in the catabolism of ectoines (Schwibbert et al., 2011; Yu et al., 2017; Hermann et al., 2020; Mais et al., 2020; Figure 1B), they will inevitably disappear from the cell when the environmental supply of ectoines has been exhausted. EnuR will consequently resume its repressor function.

Depending on the procedure to assess the phylogenomic occurrence of ectoine/5-hydroxyectoine catabolic gene clusters (Schulz et al., 2017b; Hermann et al., 2020; Mais et al., 2020), the corresponding bacteria possess between 77 and 85% juxtapositioned *enuR*-type genes. Hence, the wide-spread existence of EnuR transcriptional regulators highlights the importance of the genetic regulatory circuit that we outlined above for the transcriptional control of ectoine/5-hydroxyectoine catabolism in bacteria. At the same time, these numbers sharply pose the question how microorganisms that lack EnuR (Figure 5B) might induce and genetically control this complex metabolic pathway.

## Materials and Methods

### Chemicals and Reagents

The antibiotics gentamycin, rifampicin, and kanamycin were obtained from Serva (Heidelberg, Germany); ampicillin was purchased from Carl Roth GmbH (Karlsruhe, Germany). Anhydrotetracycline hydrochloride, desthiobiotin, and Strep-Tactin Superflow chromatography material were obtained from IBA GmbH (Göttingen, Germany). Marker proteins for size exclusion chromatography experiments were purchased from Sigma-Aldrich (Taufkirchen, Germany). Restriction endonucleases and DNA ligase were obtained from ThermoScientific (St. Leon-Rot, Germany) and used as suggested by the manufacturer. Ectoine was a kind gift from the bitop AG (Witten, Germany) and 5-hydroxyectoine was purchased from Merck (Darmstadt, Germany). γ-ADABA was purchased from abcr GmbH (Karlsruhe, Germany).

### Media and Growth Conditions

*Ruegeria pomeroyi* strains (Supplementary Table 4) were maintained on half-strength YTSS agar. For all growth experiments, the strains were cultivated in defined basal minimal medium (Baumann et al., 1971). Both media were prepared as described previously (Schulz et al., 2017b). When applicable, the antibiotics gentamycin and rifampicin, were added to liquid and solid media at a concentration of 20 μg ml^−1^. When the use of ectoine or 5-hydroxyectoine as combined carbon and nitrogen sources by *R. pomeroyi* strains was tested on basal minimal medium agar plates, the plates were supplemented with 28 mM ectoine or 28 mM 5-hydroxyectoine as sole carbon, energy and nitrogen sources. Agar plates with streaked *R. pomeroyi* strains were typically incubated at 30°C for five days.

The IBA-Stargate plasmids containing either the *enuR* (pBAS3), the *enuR*^∗^ (pBAS17), *enuR* wild-type C-terminal aminotransferase (ATD) domain (pLH17), or the *enuR*^∗^ C-terminal aminotransferase domain (pLH17) genes, were routinely maintained in the *E. coli* K-12 DH5α (Invitrogen, Karlsruhe, Germany) on LB agar plates containing ampicillin (100 μg mL^–1^). Minimal Medium A (MMA) (Miller, 1972) containing 0.5% (w/v) glucose as the carbon source, 0.5% (w/v) casamino acids (0.5%), 1 mM MgSO4, and 3 mM thiamine was used for cultivation of the *E. coli* B strain BL21 carrying plasmids pBAS3 (*enuR*^+^), pBAS17 (*enuR*^∗^), pLH17 (*enuR*-ATD) or pLH26 (*enuR*^∗^-ATD) (Supplementary Table 5) for the overproduction of the EnuR protein and its mutant derivatives (Schulz et al., 2017a,b).

### Chemical Synthesis, Purification of Ectoine Nutrient Utilization Regulator Inducers, and Metabolic Analysis

The cyclic ectoine and 5-hydroxyectoine molecules were linearized through alkaline hydrolysis as previously described (Kunte et al., 1993; Mais et al., 2020). Subsequently, the α- and γ-isomers of *N-*acetyl-L-2,4-diaminobutyric acid (α-ADABA and γ-ADABA) and hydroxy-α-ADABA were separated from other hydrolysis products of ectoine and 5-hydroxyectoine by repeated chromatography on a silica gel column (Merck silica gel 60) (Mais et al., 2020). The purity of the isolated α-ADABA and hydroxy-α-ADABA samples was at least 90% as determined by HPLC analysis. The identity and purity of these compounds was assessed by NMR spectroscopy as described by Mais et al. (2020), although we cannot exclude that minor impurities resulting from the alkaline hydrolysis of ectoines are still present in the preparations that we used for our experiments (Mais et al., 2020).

To identify metabolites derived from ectoines in *R. pomeroyi* DSS-3 cells growing in the presence if either ectoine or 5-hydroxyectoine, we carried out targeted metabolic analysis. In one set of experiments, we grew the cells in basal minimal medium with glucose (28 mM) as a carbon source and NH_4_Cl (56 mM) as a nitrogen source in the absence of ectoines (control culture). In the second set of experiments, we grew the cells in an basal medium in the absence of glucose and NH_4_Cl and provided either ectoine (56 mM) or 5-hydroxyectoine (56 mM) as sole and combined source of carbon, energy and nitrogen to the cells. In both sets of experiments, the cells were grown at 30°C in orbital shaker (20 ml culture volume in a 100 ml Erlenmeyer Flask) until the *R. pomeroyi* DSS-3 cultures reached an OD_578_ of about 1. The cells were pelleted by centrifugation, resuspended in basal medium and were then re-centrifuged. For the extraction of ectoine/5-hydroxyectoine-derived metabolites, one ml of 20% ethanol was added to the cells and they were vigorously shaken at room temperature for 30 min; cellular debris was then removed by centrifugation in table top Eppendorf centrifuge (13 000 rpm for 30 min at 4°C). The supernatant was evaporated at 50°C for at least 24 hours and the formed dry residue was re-suspended in 500 μl of double distilled water. After another centrifugation step, the supernatant was analyzed, and intracellular concentrations were estimated by assuming a volume of 0.5 μl of the cytoplasm of 1 ml *R. pomeroyi* DSS-3 cells at an OD_578_ of 1.

Separation and quantification of ectoine, 5-hydroxyectoine and its metabolites DABA, α-ADABA, γ-ADABA, hydroxy-DABA, hydroxy-α-ADABA and hydroxy-γ-ADABA in ethanolic cell extracts were conducted on a HPLC-ESI-MS system (Agilent 1,100 system with MSD1946D) using 100 mM NH_4_HCO_3_ in 90% H_2_0/10% acetonitrile as eluent. The separation column was a 250 × 2 mm i.d. Metrohm Carb 2 strong anion exchanger operated at 0.2 ml/min. The analytes were detected in selected ion modus as their positively charged H^+^ adducts. Possible interference of aspartic acid on hydroxy-DABA were checked and discarded. Calibration was performed using commercially available γ-ADABA samples. For all measurements, four independently grown *R. pomeroyi* DSS-3 cultures were used and form each of them two ethanolic extracts were prepared.

### Previously Constructed Bacterial Strains and Plasmids

The *R. pomeroyi* strain DSS-3 (Moran et al., 2004) was obtained from the German Collection of Microorganisms (DSMZ; Braunschweig, Germany), and a rifampicin-resistant [Rif^R^] derivative of this isolate (strain J470) (Todd et al., 2012) was kindly provided by J. Todd and A. Johnston (University of East Anglia, United Kingdom). *E. coli* K-12 DH5α carrying the helper plasmid pRK2013 [Kan^R^] (Figurski and Helinski, 1979) for conjugation experiments between *E. coli* and *R. pomeroyi* were also provided by these colleagues. The construction of the *R. pomeroyi eutD* mutant (ASR8) and the complete operon deletion strain ASR6 [Δ(*enuR-atf*:Gm^R^)] were described previously (Schulz et al., 2017a,b), as were plasmids pBAS3 (*enuR^+^;* wild-type) and pBAS17 (*enuR*^∗^; Lys302His) (Schulz et al., 2017a). These plasmids are derivatives of the expression vector pASG-IBA3 (IBA GmbH, Göttingen, Germany) and express *enuR* genes under the control of the anhydrotetracycline hydrochloride (AHT) responsive TetR controlled *tet* promoter carried by pASG-IBA3 and its recombinant derivatives. Both the wild-type EnuR protein and its Lys302His mutant (EnuR^∗^) carry a *Strep*-TAG-II peptide fused to their C-termini to allow affinity purification of the recombinant EnuR and EnuR^∗^ proteins from cell extracts of the *E. coli* B strain BL21 (DE3) (Schulz et al., 2017a,b).

### Newly Constructed Bacterial Strains and Plasmids

To construct a deletion of the *R. pomeroyi* chromosomal *eutE* gene, 500 bp fragments located upstream and downstream of the respective genomic region (Moran et al., 2004) were amplified by PCR using custom synthesized primers (Supplementary Table 6). A DNA fragment encompassing a gentamycin resistance cassette (Gm^R^) was amplified from plasmid p34S_Gm (Dennis and Zylstra, 1998). Using the Gibson assembly procedure (Gibson et al., 2009), the three DNA fragments were cloned into the linearized (with *Eco*RI) and dephosphorylated suicide vector pK18mobsacB (Kvitko and Collmer, 2011), which confers resistance to kanamycin. The resulting plasmid was pLH72 and carries the Δ(*eutE:*Gm^R^)1 deletion mutation (Supplementary Table 5).

Plasmids for the overproduction of the aminotransferase domains of EnuR and its Lys302His mutant derivative EnuR^∗^ were constructed via the IBA-Stargate cloning procedure as described by the manufacturer (IBA GmbH, Göttingen, Germany). Custom designed primers (Supplementary Table 6) (Microsynth AG, Balgach, Switzerland) were used to amplify the 1,110 bp aminotransferase domains (ATD) for the *enuR* and *enuR*^∗^ genes from the respective plasmids pBAS3 (*enuR*^+^) and pBAS17 (*enuR*^∗^), and were then inserted into the expression plasmid pASG-IBA3 so that recombinant proteins with a *Strep*-TAG-II affinity peptide at their carboxy-termini were produced. The resulting plasmids were pLH17 (*enuR*-CTD) and pLH26 (*enuR*^∗^-CTD), respectively (Supplementary Table 5).

Chromosomal DNA of *R. pomeroyi* strain DSS-3 was isolated as described (Marmur, 1961). The High Pure Plasmid Isolation Kit (Roche, Mannheim, Germany) was used to isolate plasmid DNA from *E. coli* strains. Chemically competent *E. coli* cells were prepared and transformed with plasmid DNA as reported (Sambrook et al., 1989). All recombinant DNA methods were carried out via routine procedures (Sambrook et al., 1989).

### Construction of a *Ruegeria pomeroyi* Chromosomal *eutE* Gene Disruption Mutant

Plasmid pLH73 [Δ(*eutE:*Gm^R^)1] (Supplementary Table 5) was conjugated by tri-parental mating into *R. pomeroyi* by mixing the *E. coli* strain PRK2015 (pRK2013 [Kan^R^]) (Figurski and Helinski, 1979), DH5α (pLH73) [Kan^R^ and Gm^R^] and the Rif^R^ *R. pomeroyi* recipient strain J470. *R. pomeroyi* J470 trans-conjugants that had received plasmid pLH73 were selected on 1/2 YTSS agar plates containing the antibiotics rifampicin and gentamycin and 10% saccharose as described (Schulz et al., 2017b). The resulting colonies were tested for their antibiotic resistance profile and Kan*^S^* Gm^R^ strains were then evaluated for the presence of the chromosomal Δ(*eutE*:Gm) deletion/insertion mutation via PCR using chromosomal DNA as the template and DNA primers listed in Supplementary Table 6 that hybridize to genomic regions flanking the *eutE* gene. The resulting *R. pomeroyi* J470-derived strain was named LHR7 [Δ(*eutE:*Gm^R^)1] (Supplementary Table 4).

### Overproduction and Purification of Ectoine Nutrient Utilization Regulator and Its Mutant Derivatives

For overproduction of the EnuR-Strep-tag-II and EnuR^∗^-Strep-tag-II recombinant proteins, cells of the *E. coli* B strain BL21 (DE3) were transformed with the appropriate overproduction plasmids pBAS3 (*enuR*^+^) or pBAS17 (*enuR*^∗^) (Supplementary Table 5). These plasmids allow the expression of the *enuR*^+^ and *enuR*^∗^ genes under the control of the *tet* promoter, a system that is controlled by the anhydrotetracycline (AHT) responsive TetR repressor whose structural gene is present on the expression plasmids (Schulz et al., 2017b). The same type of overproduction system was used to produce either the ATD from the wild-type EnuR protein (plasmid pLH17), or of the ATD from the mutant EnuR^∗^ protein (plasmid pLH26) (Supplementary Table 5). The plasmid-containing *E. coli* cells were grown at 37°C in MMA containing 0.5% casamino acids until the cultures reached an OD_578_ of about 0.5. *tet*-promoter/TetR-mediated overexpression of the various plasmid-encoded genes was triggered by adding the inducer AHT (final concentration: 0.2 mg l^–1^) to the cultures. The growth temperature of the cultures was then reduced to room temperature (about 25°C) and the cultures were subsequently incubated for additional two hours to allow overproduction of the recombinant proteins. Cells were harvested by centrifugation, resuspended in purification buffer (100 mM Tris-HCl (pH 7.5), 150 mM NaCl), lysed by passing them three to five-times through a French Pressure Cell (Aminco, Urbana, Il, United States) at 900 psi, and a cleared cell extract was obtained by centrifugation at 35,000 × *g* for 1 h at 4°C. The recombinant proteins marked with a *Strep*-TAG-II peptide were purified from the cleared cell extracts via affinity chromatography on a Strep-Tactin Superflow column as described (Schulz et al., 2017b). Strep-Tactin purified proteins were analyzed and further purified via Size-Exclusion-Chromatography (SEC) on a HiLoad 16/600 Superdex 200 pg column (GE Healthcare Europe, Freiburg, Germany), using either a buffer containing 10 mM Tris-HCl (pH 7.5) and 150 mM NaCl when the proteins were subsequently used in ligand-binding assays. The purity of all isolated proteins was assessed by sodium-dodecylsulfate (SDS) polyacrylamide gel electrophoresis (12% acrylamide). Proteins were stained and visualized with Instant*Blue* (Expedion, Cambridgeshire, United Kingdom).

### Examination of Pyridoxal-5′-Phosphate Binding to Ectoine Nutrient Utilization Regulator and Its ATD by Mass Spectrometry

Ectoine Nutrient Utilization Regulator and EnuR-ATD proteins were purified as described above. The buffer used for these preparations was 20 mM HEPES-Na pH 7.5, 115 mM NaCl, 1.2 mM CaCl_2_, 1.2 mM MgCl_2_, 2.4 mM K_2_HPO_4_. 25 μl (10 μM) of affinity purified full-length EnuR, or EnuR-ATD were treated with 10 mM NaBH_4_ (1 μl of 250 mM stock prepared freshly in 0.1 M NaOH) and incubated at room temperature for 30 min to reduce the aldimine. The NaBH_4_ reduction was quenched by acidification of the solution to pH of 5-6 with HCl and neutralized to approximately pH 7 with NaOH (Hoegl et al., 2018). These samples were immediately supplemented with 6 μl of SDS loading dye (300 mM Tris-Cl pH 6.8, 10% (w/v) SDS, 25% (v/v) β-mercaptoethanol, 25% (v/v) glycerol, 0.05% (w/v) bromo phenol blue) followed by mixing and heat treatment at 95°C for 5 min. Samples were loaded and separated on 15% polyacrylamide SDS-PAGE gels at 200 V. Gels were stained with Coomassie brilliant blue R250 [0.36% (w/v) Coomassie R250 dissolved in 46% (v/v) ethanol supplemented with 9% (v/v) glacial acetic acid] and destained with 30% (v/v) ethanol supplemented with 10% (v/v) glacial acetic acid. After destaining the protein bands corresponding to full-length EnuR, or EnuR-ATD were excised out of the gel and digested in gel by the addition of Sequencing Grade Modified Trypsin (Serva) at 37°C for 45 min, after which the supernatant was removed and further incubated at 37°C overnight. Peptides were desalted and concentrated using Chromabond C18WP spin columns (Macherey-Nagel). Finally, Peptides were dissolved in 5% (v/v) acetonitrile supplemented with 0.1% (v/v) formic acid.

Mass spectrometric analysis of the tryptic digests was performed using a timsTOF Pro mass spectrometer (Bruker Daltonic). A nanoElute HPLC system (Bruker Daltonics), equipped with an Aurora column (25 cm × 75 μm) C18 RP column filled with 1.7 μm beads (IonOpticks), was connected online to the mass spectrometer. Sample loading was performed at a constant pressure of 800 bar, and 2 μl of a 1:3 dilution of the tryptic digests in double-distilled water injected directly on the separation column. Separation was conducted at 50°C column temperature with the following gradient of water + 0.1% (v/v) formic acid (solvent A) and acetonitrile + 0.1%(v/v) formic acid (solvent B) at a flow rate of 400 nl/min: A linear increase from 2% solvent B to 17% solvent B within 60 min was followed by a linear gradient to 25% solvent B within 30 min and a linear increase to 37% solvent B in additional 10 min. Finally, solvent B was increased to 95% within 10 min and held for additional 10 min. The built-in “DDA PASEF-standard_1.1sec_cycletime” method developed by Bruker Daltonics was used for mass spectrometric measurement. Data analysis was performed using Proteome Discoverer 2.4 (ThermoScientific) with SEQUEST search engine and Byonic version 3.7.4 (Protein Metrics) using the amino acid sequences of full-length EnuR, trypsin, and keratin, as database.

### Ligand-Binding Assays With Ectoine Nutrient Utilization Regulator and Its ATD

Ligand binding assays with the purified EnuR and EnuR-ATD proteins were carried out by microscale thermophoresis (MST) (Wienken et al., 2010). All experiments were performed on a Monolith NT.115 (NanoTemper Technologies GmbH, Munich, Germany) at 21°C (red LED power was set to 80% and infrared laser power to 70%). The buffer of the purified EnuR and EnuR-ATD [in 10mM Tris- HCl (pH 7.5), 150 mM NaCl] was first exchanged with the labeling buffer of the Monolith NTTM Protein Labeling Kit RED (NanoTemper) to avoid interference of the labeling reactions with free amines in the buffer solution. Subsequent to the labeling of either EnuR, or EnuR-ATD (20 μM each) with the NT 647 dye (according to the supplier’s reaction scheme), the proteins were re-buffered into a solution buffer containing 10 mM Tris-HCl (pH 7.5), 150 mM NaCl and 0.07% Tween20. EnuR (200 nM) was titrated with α-ADABA and hydroxy-α-ADABA (starting from a ligand concentration of 1 mM). Likewise, the EnuR-ATD protein was also titrated with α-ADABA (starting from a ligand concentration of 1 mM). To determine the DNA-binding properties of EnuR, the protein was treated in the same manner and titrated with buffer containing a DNA-fragment (48 bp) harboring the presumed EnuR operator site(s) and the promoter region of the *R. pomeroyi* DSS-3 ectoine/5-hydroxyectoine importer and catabolic gene cluster (Supplementary Table 6 and Figure 8A). At least six independent MST experiments per ligand of the EnuR protein were recorded at 680 nm and analyzed using NanoTemper Analysis 1.2.009 and Origin8G software suits.

### Bioinformatic Analysis

To analyze the phylogenomic distribution of *enuR*-type genes, a recently compiled and manually curated dataset of 363 bacterial ectoine/5-hydroxyectoine catabolic gene clusters was used as a starting point (Mais et al., 2020). In this dataset, only microorganisms harboring *eutD/eutE*-genes in direct genetic neighborhood were included, as both proteins are needed to degrade ectoines (Mais et al., 2020). Accordingly, this analysis does not include ectoine/5-hydroxyectoine degradation gene clusters in which the *eutD* and *eutE* catabolic genes are not juxtapositioned [e.g., from *M. alcaliphilum* (Reshetnikov et al., 2020)]. The 363 EutD-protein sequences represented in the dataset reported by Mais et al. (2020) was retrieved from the IMG/M database (Chen et al., 2021) and represented in a tree-format visualized using the iTOL software (Letunic and Bork, 2019). Onto this EutD-protein based tree, we projected the presence of *enuR-*type genes (278 representatives) that were positioned in the immediate vicinity of ectoine/5-hydroxyectoine degradation gene clusters. Alignments of EnuR-type proteins that were obtained through IMG/JGI Web resources, were visualized with Jalview (Waterhouse et al., 2009).

A model of the presumed EnuR structure was created using the crystal structure of the *B. subtilis* GabR protein as the template (Edayathumangalam et al., 2013) and by employing the Phyre^2^ software (Kelley et al., 2015) set in the extensive mode (see Text Footnote 1). An overlay of the GabR protein and the EnuR model revealed a root mean square deviation (RMSD) of 0.86 Å (over 390 amino acids). *In silico* modelling and docking experiments for EnuR and its various ligands were carried out using Chimera (Pettersen et al., 2004) and AutoDock Vina (Trott and Olson, 2010). The definition files for the ligands α-ADABA, hydroxy-α-ADABA, γ-ADABA and DABA were created using the Schrödinger Meastro package (Release, 2017). Initial docking was performed using a wide grid setting and by allowing the positioning of the ligand all around the EnuR protein. The best solution was further optimized by multiple cycles of AutoDock Vina (Trott and Olson, 2010). Final assessment was performed by manual inspection of the interactions of each ligand within the predicted EnuR ligand binding site.

## Data Availability Statement

The original contributions presented in the study are included in the article/Supplementary Material, further inquiries can be directed to the corresponding author/s.

## Author Contributions

EB designed and supervised the study. LH planned and performed most of the experiments. LH and S-AF jointly conducted the microscale thermophoresis studies. FD performed growth experiments and extraction of metabolites. SHJS performed the *in silico* modelling and docking experiments. WS conducted the mass spectrometic analysis of EnuR proteins. AS synthesized and purified α-ADABA and hydroxy-α-ADABA and performed analysis of ectoine/5-hydroxyectoine metabolites. EB and LH wrote the manuscript with input from the other authors. All authors contributed to the article and approved the submitted version.

## Conflict of Interest

The authors declare that the research was conducted in the absence of any commercial or financial relationships that could be construed as a potential conflict of interest.

## Publisher’s Note

All claims expressed in this article are solely those of the authors and do not necessarily represent those of their affiliated organizations, or those of the publisher, the editors and the reviewers. Any product that may be evaluated in this article, or claim that may be made by its manufacturer, is not guaranteed or endorsed by the publisher.

## Footnotes

1
http://www.sbg.bio.ic.ac.uk/~phyre2/

## Abbreviations

ATD: aminotransferase domain
*K*_d_: dissociation constant
ABC transporter: ATP binding cassette transporter
TRAP transporter: tripartite ATP-independent periplasmic transporter
PLP: pyridoxal-5′-phosphate
DABA: diaminobutyric acid
α-ADABA: α-acetyldiaminobutyric acid
hydroxy-α-ADABA: hydroxy-α-acetyldiaminobutyric acid
EnuR: ectoine nutrient utilization regulator.
