# Supplementary material for: The MocR/GabR Ectoine and Hydroxyectoine Catabolism Regulator EnuR: Inducer and DNA Binding
Source: Front Microbiol. 2021 Dec 24;12:764731. doi: 10.3389/fmicb.2021.764731 (PMC8739950; doi:10.3389/fmicb.2021.764731)
Supplement: Supplementary file 1 [file Data_Sheet_1.PDF]

The MocR/GabR ectoine and hydroxyectoine catabolism regulator EnuR: Inducer and DNA binding

Lucas Hermann[1,2], Felix Dempwolff[3], Wieland Steinchen[3], Sven-Andreas Freibert[4], Sander H.J. Smits[5,6], Andreas Seubert[7], Erhard Bremer[1,3]

[1]Philipps-University Marburg, Faculty of Biology, Karl-von-Frisch Strasse 8, 35043, Marburg, Germany.
[2]Max-Planck-Institute for Terrestrial Microbiology, Department of Biochemistry and Synthetic Metabolism, Karl-von-Frisch Strasse 10, 35043, Marburg, Germany
[3]Philipps-University Marburg, SYNMIKRO Research Center, Karl-von-Frisch Strasse 14, 35043, Marburg, Germany.
[4]Philipps-University Marburg, Department of Medicine, Institute for Cytobiology and Cytopathology, and SYNMIKRO Research Center, Karl-von-Frisch Strasse 14, 35043, Marburg, Marburg, Germany.
[5]Heinrich-Heine-University, Institute of Biochemistry, Universitäts Strasse 1, 40225 Düsseldorf, Germany
[6]Heinrich-Heine-University Düsseldorf, Center for Structural Studies (CSS), Faculty of Biochemistry, Universitäts Strasse 1, 40225, Düsseldorf, Germany.
[7]Philipps-University Marburg, Faculty of Chemistry, Hans-Meerwein Strasse 4, 35043, Marburg, Germany.

Running title: Genetic control of ectoine/hydroxyectoine utilization

ORCID

Lucas Hermann: https://orcid-org/0000-0001-6684-1644
Felix Dempwolff: https://orcid-org/0000-0002-7788-8445
Wieland Steinchen: https://orcid-org/0000-0003-2990-3660
Sven-Andreas Freibert: https://orcid-org/0000-0002-8521-2963
Sander H.J. Smits: https://orcid-org/0000-0003-0780-9251
Andreas Seubert: https://orcid-org/0000-0002-7398-363x
Erhard Bremer: https://orcid-org/0000-0002-2225-7005

For correspondence:

Dr. Erhard Bremer, Philipps-University Marburg, SYNMIKRO Research Center, Karl-von-Frisch Strasse 14, 35043, Marburg, Germany. Phone: (+49)-6421-2821529; Fax: (+49)-6421-2822229; E-Mail: bremer@staff.uni-marburg.de

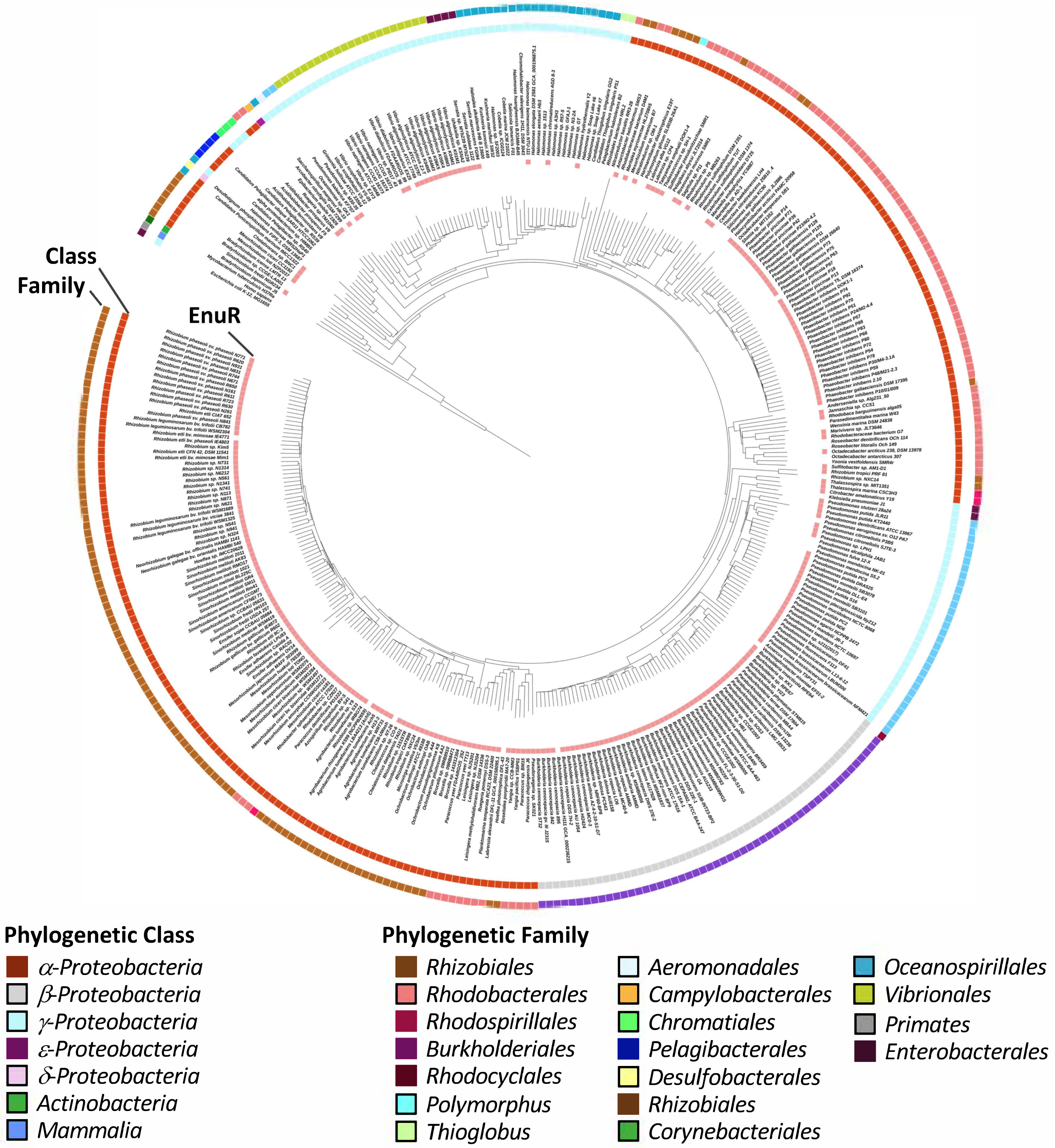

Supplementary Figure S1. Phylogenomics of EnuR-type proteins.

A phylogenetic tree of the ectoine/-5-hydroxyectoine hydrolase EutD was established using the iTol server (Letunic and Bork, 2019). The tree of 364 EutD-type proteins (Mais et al., 2020) is rooted with *Escherichia coli* and *Homo sapiens* aminopeptidases (Bradshaw et al., 1998;Wilce et al., 1998). The phylogenetic groups of microorganisms possessing EutD-type ectoine/5-hydroxyectoine hydrolases are highlighted in the two outer circles and the ectoine/5-hydroxyectoine catabolic gene clusters were analyzed for the presence of EnuR homologues in their vicinity as indicated in the inner circle.

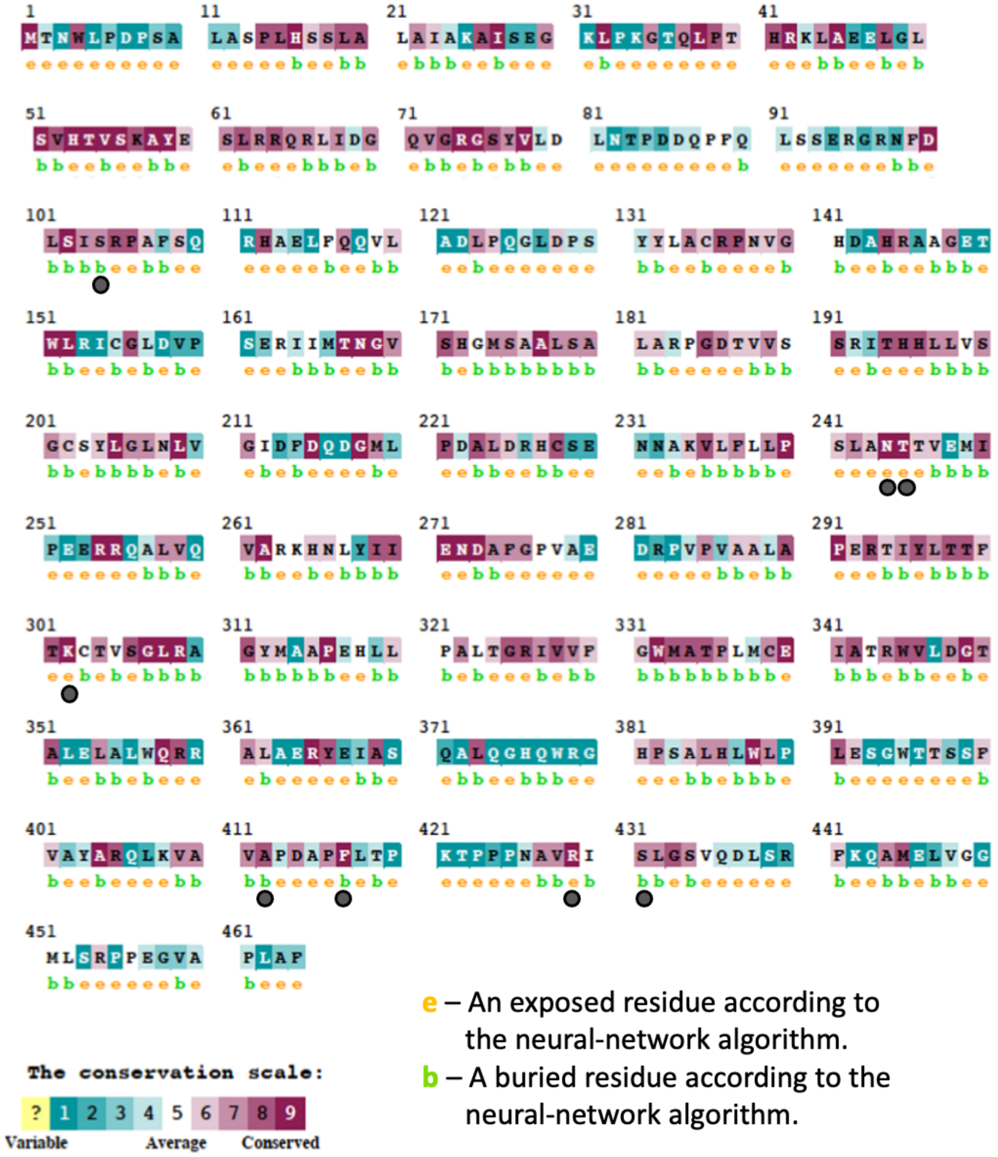

Supplementary Figure S2. Evolutionary conservation of EnuR-type proteins.

The degree of amino acid conservation of individual amino acids of the EnuR protein of *Ruegeria pomeroyi* DSS-3 using the ConSurf server (Berezin et al., 2004). The amino acid sequences of 278 EnuR-like proteins were used in an alignment to derive a conservation matrix. The amino acid sequence of the EnuR *R. pomeroyi* DSS-3 query protein (SPO1148) (Moran et al., 2004) is displayed with the degree of evolutionary conservation at position in the protein chain. Each site is color-coded according to the degree of conservation. The first row below the sequence lists the predicted burial status of the site (see legend) (Berezin et al., 2004). Black dots mark the amino-acids determined in the *in-silico* modelling and docking experiments to be crucial for ligand-binding.

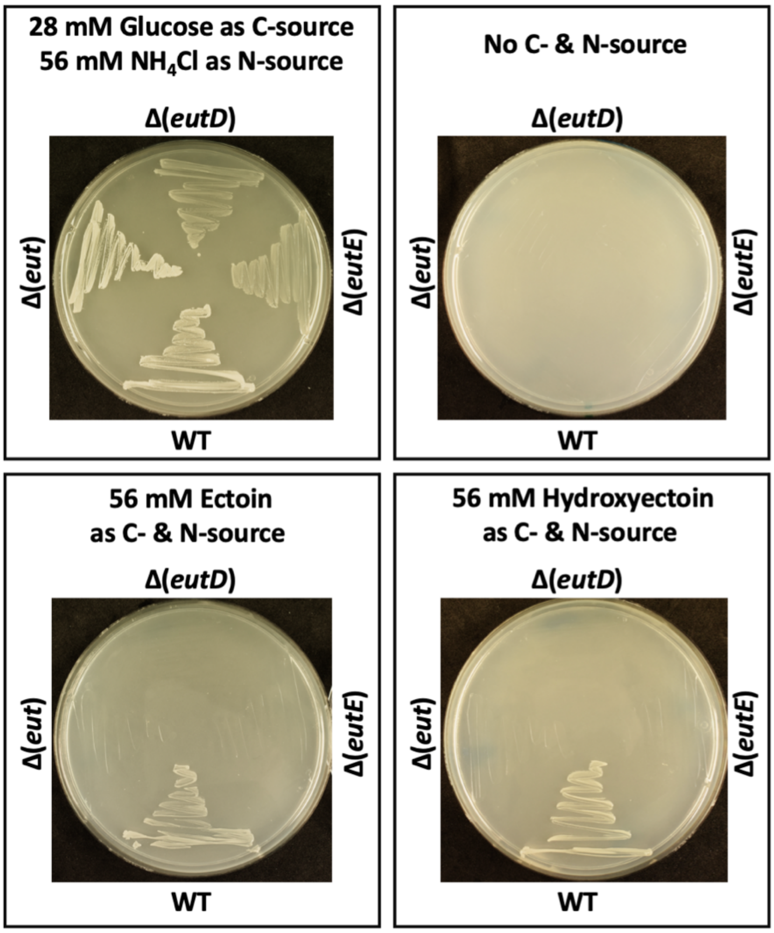

Supplementary Figure S3. Utilization of ectoines by *R. pomeroyi* DSS-3 as nutrients.

Growth of the *R. pomeroyi* wild-type strain J470 and its mutant derivatives [ASR6 D(*eut::gm* ^{R}), ASR8 D(*eutD*::*gm*^{R}), and LHR7 D(*eutE*::*Gm*^{R})] on basal minimal medium agar plates containing 28 mM ectoine or 5-hydroxyectoine when used either as sole carbon or nitrogen source. The D(*eut::gm*^{R}) allele removes the entire ectoine/5-hydroxyectoine importer and catabolic gene cluster (from *enuR to atf*; see **Figure 1A**). Colonies were picked from basal medium agar plates containing glucose and NH_4Cl as carbon and nitrogen sources and streaked onto basal minimal agar plates containing the indicated carbon and nitrogen sources. The agar plates were incubated at 30° C for five days.

Supplementary Table S1. Phylogenetic groups of ectoine-consumers and distribution of EnuR-like proteins.

Phylogenetic group	Number of organisms	Organisms possessing EnuR like proteins	Percentage of organisms possessing EnuR [%]
Rhizobiales	117	103	88.0
Rhodobacteraceae	89	70	78.7
Burkholderiales	52	50	96.2
Pseudomonales	33	29	87.9
Oceanospirillales	25	5	20.0
Vibrionales	22	12	54.5
Minor groups	25	9	34.6
Total	363	278	76.6

Using computational tools provided via the IMG/M web-server (Chen et al., 2021), 363 microbial genome sequences (out of 8 850 inspected genome sequences) contained juxtapositioned *eutD/eutE* pairs in their ectoine/5-hydroxyectoine catabolic gene clusters (Mais et al., 2020). The taxonomic association of the corresponding microorganisms were assessed and the presence of *enuR*-type genes in the immediate vicinity of the ectoine/5-hydroxyectoine catabolic gene clusters was tabulated.

Supplementary Table S2. Docking of inducers into the presumed effector binding site of EnuR.

Inducer molecule	Binding energy (kCal/ mol)	Residues involved in hydrogen bonding
Hydroxy-α-ADABA	-7.3	Asn244, Thr245, Phe417, Ser431, Ser104
α-ADABA	-5.9	Asn244, Ser104, Ser431, Thr245
DABA	-3.8	Ser431

Results of the docking studied using AutoDock Vina (Trott and Olson, 2010) showing the predicted free energy change upon ligand-binding by the *R. pomeroyi* DSS-3 EnuR protein, and tabulation of the amino acids predicted to be involved in inducer-binding.

Supplementary Table S3. Conservation of amino acids of EnuR predicted to be involved in α-ADABA and hydroxy-α-ADABA binding.

Residue	Conservation	Functional replacement
Ser104	274/278	3 Thr; 1 Ala
Asn244	260/278	1 Ser; 12 Gly; 5 Ala
Thr245	196/278	2 Tyr; 2 Gln; 4 Arg; 4 Asn; 57 Met; 4 Leu; 3 Lys; 2 His; 4 Phe
Lys302	278/278	
Ala412	148/278	123 Leu; 3 Met; 4 Cys
Phe417	278/278	
Arg429	278/278	
Ser431	133/278	7 Ala; 135 Cys; 3 Asn

The amino acid sequences of 278 EnuR-type proteins were aligned with Jalview (Waterhouse et al., 2009) and the conservation of those amino acids implicated by our modelling and docking studies for the binding of the inducer molecules α-ADABA and hydroxy-α-ADABA were assessed.

Supplementary Table S4. Strains used in this study.

Strain	Genotype or description	Source or reference
Escherichia coli DH5a	Used for routine cloning purposes	Invitrogen, Karlsruhe, Germany
Escherichia coli BL21 (DE3)	Strain, used for overexpression	Stratagene, La Jolla, CA
Ruegeria pomeroyi DSS-3	Wild-type strain	(Moran et al., 2004)
Ruegeria pomeroyi J470[a]	Rif^R derivative of the wild-type strain	(Todd et al., 2012)
Ruegeria pomeroyi ASR6[b]	*R. pomeroyi* J470 Δ(*enuR-atf::gm*)1	(Schulz et al., 2017a)
Ruegeria pomeroyi ASR8[b]	*R. pomeroyi* J470 Δ(*eutD::gm*)1	(Schulz et al., 2017a)
Ruegeria pomeroyi LHR7[b]	*R. pomeroyi* J470 Δ(*eutE::gm*)1	This study

[a]Rif^R: Resistant against the antibiotic rifampicin.

[b]*gm*: Genetic determinant conferring resistance against the antibiotic gentamycin.

Supplementary Table S5. Plasmids used in this study.

Plasmid	Genotype or description	Source or reference
pRK2013[a]	Helper plasmid for tri-parental mating, Kan^R	(Figurski and Helinski, 1979)
pK18mobsacB	Suicide vector for *R. pomeroyi*, Kan^R	(Kvitko and Collmer, 2011)
p34S-*gm*[c]	Plasmid carrying a gentamicin (Gm^R) resistance cassette	(Dennis and Zylstra, 1998)
pLH73	pK18mobsacB with flanking regions of the *eutE*-gene, interrupted with a Gm^R cassette, Kan^R	This study
pEntry51	Cloning vector for IBA-Stargate cloning	IBA GmbH, Göttingen, Germany
pASG-IBA3	*E. coli* expression vector carrying a TetR-controlled and anhydrotetracyclin-responsive *tet* promoter	IBA GmbH, Göttingen, Germany
pBAS3[d]	pASG-IBA3 with synthetic, codon optimized *enuR* gene	(Schulz et al., 2017b)
pBAS17[d]	pBAS3 with codon exchange mutation (AAA/CAT) in the codon-optimized *enuR* leading to the replacement of Lys-302 with a His residue	(Schulz et al., 2017a)
pLH17[d]	pASG-IBA3 with synthetic, codon optimized sole aminotransferase domain of the *enuR* gene	This study
pLH26[d]	pLH17 with codon exchange mutation (AAA/CAT) in the codon-optimized *enuR* gene leading to the replacement of Lys-302 with a His residue	This study

[a]Kan^R: Resistant against the antibiotic kanamycin.

[b]*gm*: Genetic determinant conferring resistance against the antibiotic gentamycin.

[c]The *R. pomeroyi* DSS-3 *enuR* gene (or a segment thereof) carried by these plasmids was codon-optimized for enhanced expression in *E. coli* (Schulz et al., 2017b). The DNA-sequence of this synthetic gene is available in GenBank under accession number KU891821.

Supplementary Table S6. Oligonucleotides used in this study.

Primer	Sequence	Reference, description
ATD_pEntry_fw	AAGCTCTTCAATGCGTAATTTTGATCTGAGCA TTAGCCG	IBA-Stargate cloning of the ATD of *Ruegeria pomeroyi* EnuR
ATD_pEntry_rev	AAGCGGCTCTTCTCCCAAGCTCTTCACCCAAA TGCCAG	IBA-Stargate cloning of the ATD of *Ruegeria pomeroyi* EnuR
L263_fw	GGTCGGCGGCATGCTG	EMSA-fragments for the *uehA*-operator region
L229_rev_dye	GGTTTCCTCCCAAATGTCATGGG	EMSA-fragments for the *uehA*-operator region
Δ*eutE*_F1_fw	ACAGCTATGACATGATTACGCGCATCTGACCT GGGACGAT	Construction of plasmid pLH73
Δ*eutE*_F1_rev	ttcgagctcgAGTCCTTCACGAACATCTTGCGCG G	Construction of plasmid pLH73
Δ*eutE*_gm_fw	GTGAAGGACTcgagctcgaattgacataagcctgtt	Construction of plasmid pLH73
Δ*eutE_gm*_rev	GGTCCGCCTCtgttaggtggcggtacttgggt	Construction of plasmid pLH73
Δ*eutE*_F2_fw	ccacctaacaGAGGCGGACCCATGCA	Construction of plasmid pLH73
Δ*eutE*_F2_rev	ATCCCCGGGTACCGAGCTCGGCTGGCGCCGT CACT	Construction of plasmid pLH73
MST BS WT fw	TAACATTGTCGCGCGACAATAAAAAAATTGA CATGCAGTACAATTCCC	Fragment for MST[a]
MST BS WT rev	GGGAATTGTACTGCATGTCAATTTTTTTATTG TCGCGCGACAATGTTA	Fragment for MST[a]

[a]MST: micoscale thermophoresis

References

Berezin, C., Glaser, F., Rosenberg, J., Paz, I., Pupko, T., Fariselli, P., Casadio, R., and Ben-Tal, N. (2004). ConSeq: the identification of functionally and structurally important residues in protein sequences. *Bioinformatics* 20**,** 1322-1324.

Bradshaw, R.A., Brickey, W.W., and Walker, K.W. (1998). N-terminal processing: the methionine aminopeptidase and N alpha-acetyl transferase families. *Trends Biochem. Sci.* 23**,** 263-267.

Chen, I.A., Chu, K., Palaniappan, K., Ratner, A., Huang, J., Huntemann, M., Hajek, P., Ritter, S., Varghese, N., Seshadri, R., Roux, S., Woyke, T., Eloe-Fadrosh, E.A., Ivanova, N.N., and Kyrpides, N.C. (2021). The IMG/M data management and analysis system v.6.0: new tools and advanced capabilities. *Nucleic Acids Res.* 49**,** D751-D763.

Dennis, J.J., and Zylstra, G.J. (1998). Improved antibiotic-resistance cassettes through restriction site elimination using Pfu DNA polymerase PCR. *Biotechniques* 25**,** 772-774, 776.

Figurski, D.H., and Helinski, D.R. (1979). Replication of an origin-containing derivative of plasmid RK2 dependent on a plasmid function provided in trans. *Proc. Natl. Acad. Sci U S A* 76**,** 1648-1652.

Kvitko, B.H., and Collmer, A. (2011). Construction of *Pseudomonas syringae* pv. tomato DC3000 mutant and polymutant strains. *Methods Mo.l Biol.* 712**,** 109-128.

Letunic, I., and Bork, P. (2019). Interactive Tree Of Life (iTOL) v4: recent updates and new developments. *Nucleic Acids Res.* 47**,** W256-W259.

Mais, C.-N., Hermann, L., Altegoer, F., Seubert, A., Richter, A.A., Wernersbach, I., Czech, L., Bremer, E., and Bange, G. (2020). Degradation of the microbial stress protectants and chemical chaperones ectoine and hydroxyectoine by a bacterial hydrolase-deacetylase complex. *J. Biol. Chem.* 295**,** 9087-9104.

Moran, M.A., Buchan, A., Gonzalez, J.M., Heidelberg, J.F., Whitman, W.B., Kiene, R.P., Henriksen, J.R., King, G.M., Belas, R., Fuqua, C., Brinkac, L., Lewis, M., Johri, S., Weaver, B., Pai, G., Eisen, J.A., Rahe, E., Sheldon, W.M., Ye, W., Miller, T.R., Carlton, J., Rasko, D.A., Paulsen, I.T., Ren, Q., Daugherty, S.C., Deboy, R.T., Dodson, R.J., Durkin, A.S., Madupu, R., Nelson, W.C., Sullivan, S.A., Rosovitz, M.J., Haft, D.H., Selengut, J., and Ward, N. (2004). Genome sequence of *Silicibacter pomeroyi* reveals adaptations to the marine environment. *Nature* 432**,** 910-913.

Schulz, A., Hermann, L., Freibert, S.-A., Bönig, T., Hoffmann, T., Riclea, R., Dickschat, J.S., Heider, J., and Bremer, E. (2017a). Transcriptional regulation of ectoine catabolism in response to multiple metabolic and environmental cues. *Env. Microbiol.* 19**,** 4599-4619.

Schulz, A., Stöveken, N., Binzen, I.M., Hoffmann, T., Heider, J., and Bremer, E. (2017b). Feeding on compatible solutes: a substrate-induced pathway for uptake and catabolism of ectoines and its genetic control by EnuR. *Environ. Microbiol.* 19**,** 926-946.

Todd, J.D., Kirkwood, M., Newton-Payne, S., and Johnston, A.W. (2012). DddW, a third DMSP lyase in a model Roseobacter marine bacterium, *Ruegeria pomeroyi* DSS-3. *ISME J* 6**,** 223-226.

Trott, O., and Olson, A.J. (2010). AutoDock Vina: improving the speed and accuracy of docking with a new scoring function, efficient optimization, and multithreading. *J. Comput. Chem.* 31**,** 455-461.

Waterhouse, A.M., Procter, J.B., Martin, D.M., Clamp, M., and Barton, G.J. (2009). Jalview Version 2--a multiple sequence alignment editor and analysis workbench. *Bioinformatics* 25**,** 1189-1191.

Wilce, M.C., Bond, C.S., Dixon, N.E., Freeman, H.C., Guss, J.M., Lilley, P.E., and Wilce, J.A. (1998). Structure and mechanism of a proline-specific aminopeptidase from *Escherichia coli*. *Proc Natl Acad Sci U S A* 95**,** 3472-3477.
